# The Rho GTPase Rnd1 inhibits epithelial–mesenchymal transition in hepatocellular carcinoma and is a favorable anti-metastasis target

**DOI:** 10.1038/s41419-018-0517-x

**Published:** 2018-04-30

**Authors:** Cheng-Dong Qin, De-Ning Ma, Shi-Zhe Zhang, Ning Zhang, Zheng-Gang Ren, Xiao-Dong Zhu, Qing-An Jia, Zong-Tao Chai, Cheng-Hao Wang, Hui-Chuan Sun, Zhao-You Tang

**Affiliations:** 1 Department of Breast Surgery, Zhejiang Cancer Hospital, 1 Banshan East Road, Hangzhou, 310022 China; 2 Department of Colorectal Cancer Surgery, Zhejiang Cancer Hospital, 1 Banshan East Road, Hangzhou, 310022 China; 3 Liver Cancer Institute, Zhongshan Hospital, Fudan University, Key Laboratory of Carcinogenesis and Cancer Invasion (Fudan University), Ministry of Education, 179 Fenglin Road, Shanghai, 200032 China; 4 Liver Surgey Department, Shanghai Cancer center, 270 Dong’an Road, Shanghai, 200032 China; 5 Department of Hepatobiliary Surgery, First Affiliated Hospital of Xi’an Jiaotong University, Xi’an, 710061 China; 6 Department of Hepatic Surgery, Eastern Hepatobiliary Surgery Hospital, Second Military Medical University, Shanghai, 200433 China

## Abstract

Rnd1, a member of Rho GTPases, was found to be downregulated in human malignancies and downregulation of Rnd1 promotes tumor invasion via various mechanisms. However, the role of Rnd1 in hepatocellular carcinoma (HCC) progression remains unclear. In this study, our results demonstrated that Rnd1 was downregulated in HCC cells and in human HCC tissues. Low expression of Rnd1 was associated with aggressive clinic-pathologic characteristics, such as vascular invasion, and poor prognosis in patients who underwent curative surgery for HCC. Overexpression of Rnd1-suppressed cell growth, migration, invasion, and EMT processes in vitro and in vivo. Furthermore, Rnd1 blocked HCC progression by restricting EMT process through inhibition of the Raf/MEK/ERK cascade, and this was correlated with a reduction in RhoA activity. Combination of Rnd1 overexpression with sorafenib, a Raf signaling pathway inhibitor, showed a more potent inhibition on HCC metastasis. Moreover, epigenetic inhibitors (5-Aza and SAHA) increased the expression of Rnd1, and potentiated sorafenib-induced toxicity in HCC cells. In a conclusion, Rnd1-suppressed EMT-mediated metastasis of HCC by reducing the activity of the RhoA/Raf/MEK/ERK signaling pathway, functioning as a favorable anti-metastasis target for HCC patients. Rnd1 overexpression in combination with sorafenib may result in enhanced anti-metastasis efficacy in HCC.

## Introduction

Liver cancer, mostly hepatocellular carcinoma (HCC), is the fifth most prevalent malignance and the second-leading cause of cancer-related mortality among men worldwide^1^. Surgical resection is the primary treatment for HCC. However, the 5-year recurrence rate is as high as 70%, primarily due to intrahepatic and extrahepatic metastasis^2^. Decades of research have brought insight into the progression of tumors with the identification of a set of genes correlated with metastasis and exploration of therapeutic targets to prolong patients’ survival^3,4^. However, there are still many unexplored factors in the progression of HCC metastasis. To further reveal the driving events of HCC metastasis is of great significance.

Epithelial–mesenchymal transition (EMT) is a critical step in invasion-metastasis cascades^5^. During this process, tumor cells reversibly transform from an epithelial phenotype to a mesenchymal phenotype, followed by enhanced cell invasive capabilities. And EMT is characterized by downregulation of the epithelial marker, E-cadherin, and upregulation of mesenchymal markers, such as N-cadherin and vimentin^6^. Our previous studies had revealed that EMT played a key role in chemo-resistance of HCC and could be modulated by microRNAs, genes, and drugs^7,8^.

Rho GTPases belongs to the family of small G proteins, which acts as molecular switches by cycling between active state (GTP-bound) and inactive state (GDP-bound). Only in the active state, Rho GTPases could combine to a wide variety of targeted proteins to generate a response in the modulation of cytoskeleton dynamics and gene transcription^9^. Through this process, Rho GTPases play a pivotal role in the regulation of cellular physiological functions, such as cell polarity, adhesion, locomotion, and invasion, as well as cell proliferation and survival^10^. Moreover, the crosstalk among different Rho GTPases has been shown to regulate the process of EMT^11^. Deregulation of Rho GTPases has also been validated to be intricately involved in tumor progression^10^. As a member of the Rho family of GTPases, Rnd1 is located at chromosome 12q12-q13, the deletion of which is regularly observed in pancreatic cancer and adenoid cystic carcinoma^12,13^. Besides, it had been shown that inactivation of Rnd1 could drive breast cancer initiation and progression^14^. Nevertheless, whether Rnd1 plays a role in HCC progression remains undetermined. In the present study, we aimed to investigate the epigenetic regulation and the underlying mechanisms of Rnd1 in HCC.

## Results

### Downregulation of Rnd1 is associated with aggressive clinic pathological features and poor prognosis in HCC

**Fig. 1:**
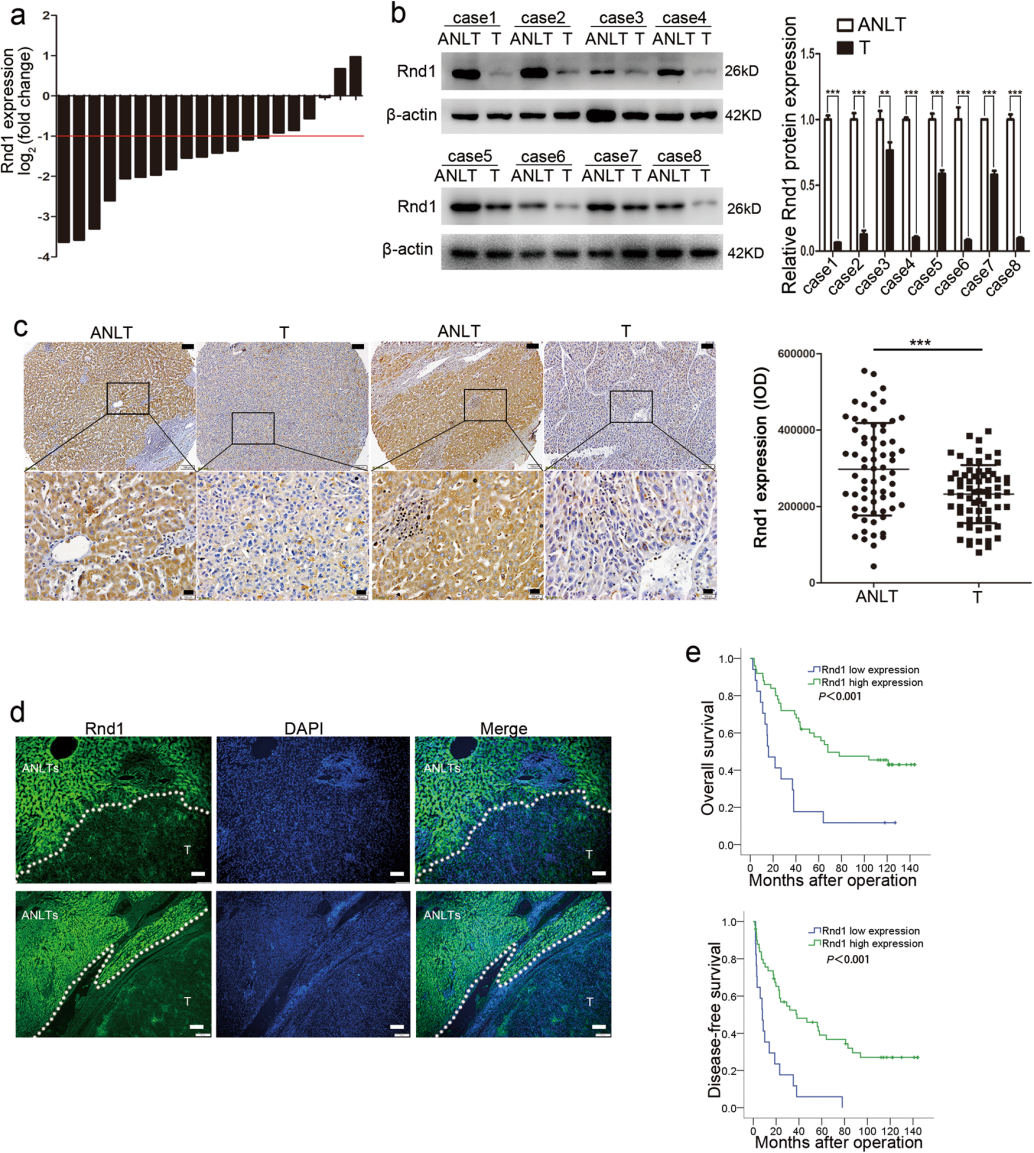
Rnd1 expression is downregulated in HCC and predicts poor prognosis. **a** Rnd1 mRNA expression in 20 paired HCC tissues (T) and adjacent non-tumor liver tissues (ANLTs). Expression level of Rnd1 was normalized to GAPDH. Fold changes were analyzed using the formula: 2^−(∆∆ CT [HCC/ANLT])^. Red line indicates fold change of Rnd1 equal to 2. **b** Protein expression of Rnd1 in select HCC tissues (T; *n* = 8) and adjacent non-tumor liver tissues (ANLTs; *n* = 8). Densitometry of western blot in panel. Data represent means ± S.D. of three independent experiments, significant difference was tested by Student’s *t*-test. **P < 0.01, ***P < 0.001. **c** Representative IHC images of Rnd1 expression in HCC tissues (T) and adjacent non-tumor liver tissues (ANLTs). Scale bar: 100 μm (upper), 20 μm (lower). Rnd1 expression was quantitated based on integrated optical density (IOD). Results were expressed as mean ± S.D., *n* = 67 patients. Significant difference was tested by Student’s *t*-test. ***P < 0.001. **d** Representative IF images of Rnd1 expression in HCC tissues (T) and adjacent non-tumor liver tissues (ANLTs); boundaries between T and ANLT tissue are indicated by a dashed line. Scale bar: 100 μm. **e** Kaplan–Meier analysis indicates that low expression of Rnd1 is correlated with poor overall survival and disease-free survival in 67 cases of HCC patients. **P < 0.01

The mRNA expression level of Rnd1 was compared in 20 paired HCC tissue and adjacent non-tumor liver tissue (ANLT). The results showed that the mean level of Rnd1 mRNA in 14 HCC cases was significantly lower (more than twofold; i.e., log2 [fold change] > 1) than that in the ANLTs (70.0%; Fig. 1a). Consistent with these findings, Rnd1 protein level was decreased in HCC tissues compared with ANLT by western blot, immunohistochemistry, and immunofluorescence assay (Fig. 1b–d).

To illustrate the clinical relevance of Rnd1 expression in HCC, the cutoff finder software was used to determine the optimal cutoff points to separate patients into high and low expression group, based on integral optical density (IOD). According to this criteria, associations between Rnd1 expression and clinicopathologic characteristics of HCC were evaluated, the results showed that patients with low Rnd1 expression had a large tumor size, high incidence of microvascular invasion, and poor cell differentiation in tumor tissue (Supplementary table 1). Similarly, HCC cases with vascular invasion had lower expression of Rnd1 than those without vascular invasion in the TCGA data sets (*P* = 0.019, Supplementary Figure 1). Survival analysis suggested that patients with low Rnd1 expression in HCC tissue had shorter overall survival (OS) and disease-free survival (DFS) time than those with high expression (Fig. 1e). Multivariate analysis identified Rnd1 expression as an independent risk factor for OS and DFS (Table 1). These results suggested that low Rnd1 expression was associated with invasive characteristics and poor prognosis in HCC patients.

**Table 1:** Univariate and multivariate analyses of risk factors associated with overall survival and disease-free survival of HCC patients in training cohort

Variables		Overall Survival				Disease-free survival			
	*n*	Univariate analysis		Multivariate analysis		Univariate analysis		Multivariate analysis	
		HR(95% CI)	*P*	HR(95% CI)	*P*	HR(95% CI)	*P*	HR(95% CI)	*P*
*Sex*									
Male	58	1	0.433		NA	1	0.385		NA
Female	9	0.689(0.271–1.751)				0.685(0.292–1.609)			
*Age, years*									
≤60	53	1	0.574		NA	1	0.321		NA
>60	14	1.226(0.603–2.494)				1.389(0.725–2.660)			
*HBV*									
Negative	8	1	**0.040**	1	NS	1	0.216		NA
Positive	59	4.443(1.072–18.414)		2.588(0.589–11.017)		1.716(0.729–4.041)			
*Liver cirrhosis*									
Absence	8	1	0.051		NA	1	0.454		NA
Presence	59	4.128(0.996–17.105)				1.386(0.590–3.259)			
*Tumor size, cm*									
≤5 cm	47	1	** <0.001**	1	**0.003**	1	**0.004**	1	**0.042**
>5 cm	20	3.432(1.833–6.426)		2.919(1.443–5.903)		2.383(1.320–4.302)		1.934(1.024–3.655)	
*AFP, ng/ml*									
<20	27	1	0.646		NA	1	0.634		NA
≥20	40	1.153(0.625–2.125)				1.146(0.653–2.013)			
*Capsulation formation formation formation*									
Absence	31	1	0.162		NA	1	0.055		NA
Presence	36	0.652(0.357–1.188)				0.582(0.335–1.012)			
*Microvascular invasion*									
Presence	12	1	** <0.001**	1	NS	1	** <0.001**	1	NS
Absence	55	6.204(2.976–12.933)		2.050(0.773–5.434)		4.893(2.378–10.069)		1.641(0.648–4.156)	
*Edmondson-Steiner grade*									
I & II	43	1	**0.001**	1	NS	1	** <0.001**	1	**0.008**
III & IV	24	2.731(1.487–5.015)		2.117(0.967–4.634)		3.235(1.809–5.785)		2.423(1.262–4.652)	
*BCLC stage*									
0 & A	9	1	**0.020**	1	**0.042**	1	0.195		NA
B & C	58	10.589(1.453–77.186)		8.104(1.081–60.767)		1.760(0.748–4.144)			
*TNM stage*									
Early (I & II)	59	1	0.083		NA	1	0.549		NA
Late (III & IV)	8	1.876(0.921–3.822)				1.247(0.606–2.566)			
*Rnd1 expression*									
Low	17	1	**0.001**	1	**0.038**	1	**<0.001**	1	**0.044**
High	50	0.318(0.167–0.607)		0.447(0.209–0.955)		0.297(0.160–0.550)		0.450(0.207–0.978)	

*HR* hazard risk ratio, *CI* confidence interval, *NA* not applicable, *NS* not significant

^*^Significant results (*P*<0.05) are given in bold

### Downregulation of Rnd1 facilitates growth, migration, and metastasis of HCC cells in vitro and in vivo

**Fig. 2:**
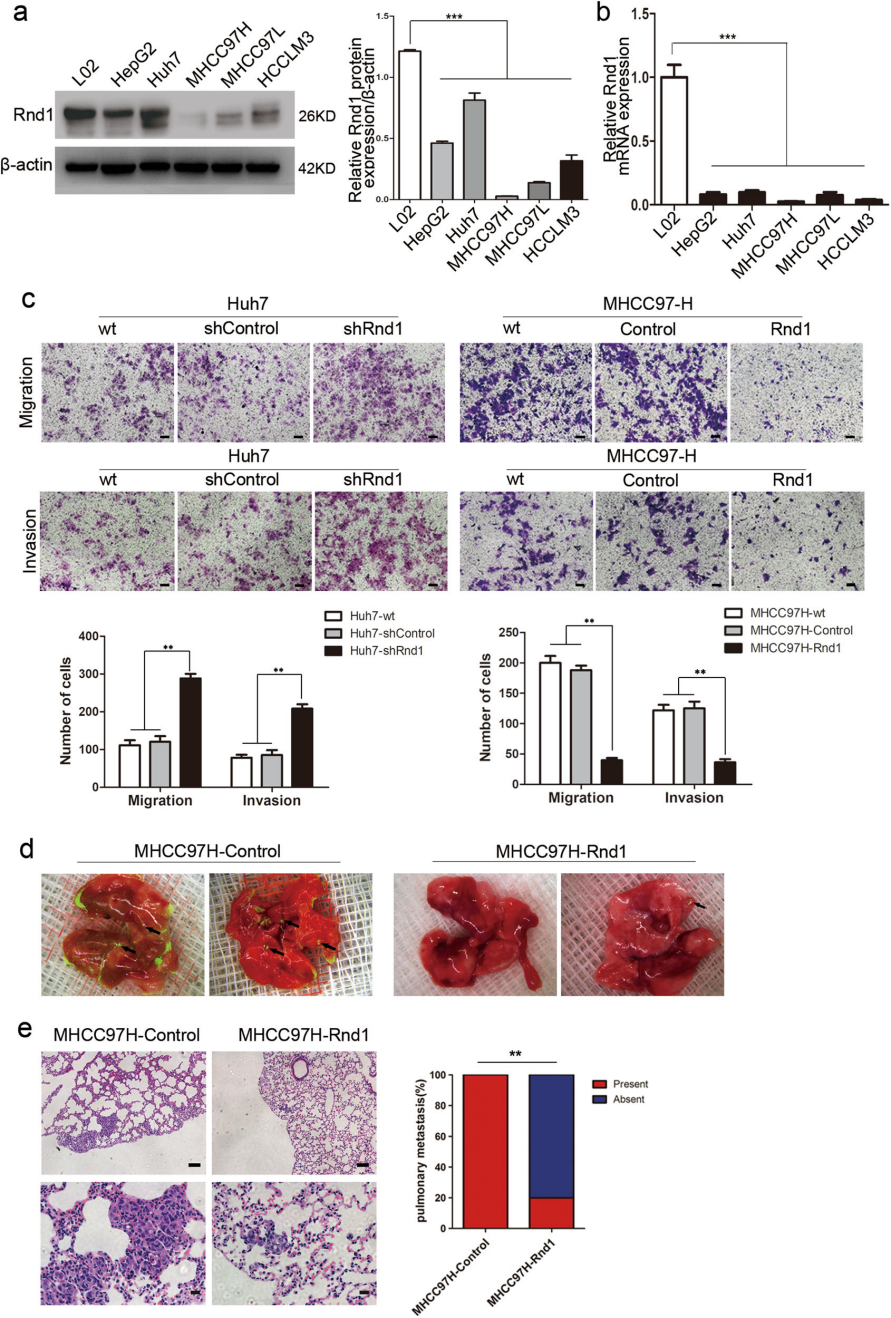
Attenuated Rnd1 expression promotes the migration and invasion of HCC cells in vitro and in vivo. **a**, **b** Western blot and Real-time PCR analysis of the Rnd1 protein and mRNA expression, respectively, in one normal liver cell line (L02) and five HCC cell lines (Huh7, HepG2, MHCC97L, MHCC97H, and HCCLM3). Panels represent mean ± S.D. (*n* = 3) from three independent experiments plated in triplicate. Significance was determined using one-way ANOVA. ***P < 0.001. **c** The impact of Rnd1 knockdown and overexpression on cell migration and invasion, as evaluated by transwell assays with or without Matrigel. Representative images of migrated or invasive cells are shown. Scale bar: 100 μm. The number of cells passed through the membrane was counted and compared in the graphs below, the results are expressed as the mean ± S.D. (*n* = 3) of three independent experiments, significance was determined using one-way ANOVA. **P < 0.01. **d** Representative fluorescence images of lungs derived from orthotopic xenograft tumor models. **e** Representative HE staining images for lung metastasis. Scale bar: 100 μm (upper), 20 μm (lower). The percentage of mice with or without metastatic nodules in the lungs was calculated and compared (5 mice in each group). Significance was determined using *χ*^2^ test. **P < 0.01

As Rnd1 expression is associated with microvascular invasion in human HCC, we focused on the effects of Rnd1 on migration and invasion of HCC cells. First, we measured the mRNA and protein expression of Rnd1 by real-time PCR and western blot in a normal liver cell line (L02) and in a series of HCC cell lines with varying metastatic potential (MHCC97H, HCCLM3, MHCC97L, HepG2, and Huh7)^15^. Compared with L02, Rnd1 expression was lower in the HCC cells (Fig. 2a, b). We used short hairpin RNA (shRNA) to interfere the expression of Rnd1 in the Huh7 cell line (termed Huh7-shControl/Huh7-shRnd1; Supplementary Figure 2a) and used complementary DNA (cDNA) to stably overexpress Rnd1 in MHCC97H cell line (termed MHCC97H-Control/MHCC97H-Rnd1; Supplementary Figure 2b). Rnd1 showed mainly cytoplasmic expression in MHCC97H cells by immunofluorescence (Supplementary Figure 2c).

A transwell assay with or without Matrigel showed that migration and invasion were increased in Huh7-shRnd1 cells, and were decreased in MHCC97H-Rnd1 cells, compared to corresponding control groups (Fig. 2c). Furthermore, wound-healing assays demonstrated that Rnd1 expression is reversed related with the speed of wound closure (Supplementary Figure 3). These results suggested that Rnd1 negatively regulated tumor migration and invasion of HCC cells in vitro. Additionally, Rnd1 also suppressed cell proliferation and arrested the cell cycle in the G2/M phase (Supplementary Figure 4a-d), but had no effect on the apoptosis of HCC cells in vitro (Supplementary Figure 4e). Moreover, in order to reduce the possibility of off-target effects, we designed another three shRNA plasmids to interfere Rnd1 expression in Huh7 cells. Two working shRNA plasmids inhibited Rnd1 expression and their effects of knockdown could be overcome by expressing Rnd1 cDNA (Supplementary Figure 5a, b). Besides, the transfection of them also promoted the proliferation, migration and invasion of Huh7 cells in vitro (Supplementary Figure 5c-e).

We established an orthotopic xenograft tumor models to verify the effects of Rnd1 on HCC progression in vivo. Forty days after tumor incubation, the tumor volume in the Huh7-shRnd1 group was larger than that of the Huh7-control group (*P* = 0.01; Supplementary Figure 6a), and the tumor volume in MHCC97H-Rnd1 group was smaller than that of the MHCC97H group (*P* < 0.01; Supplementary Figure 6b). All mice in the MHCC97H-control group developed lung metastasis, whereas only one mouse in the MHCC97H-Rnd1 group (Fig. 2d, e). These results indicated that Rnd1-suppressed HCC growth and metastasis in vivo.

### Downregulation of Rnd1 induces EMT of HCC cells

**Fig. 3:**
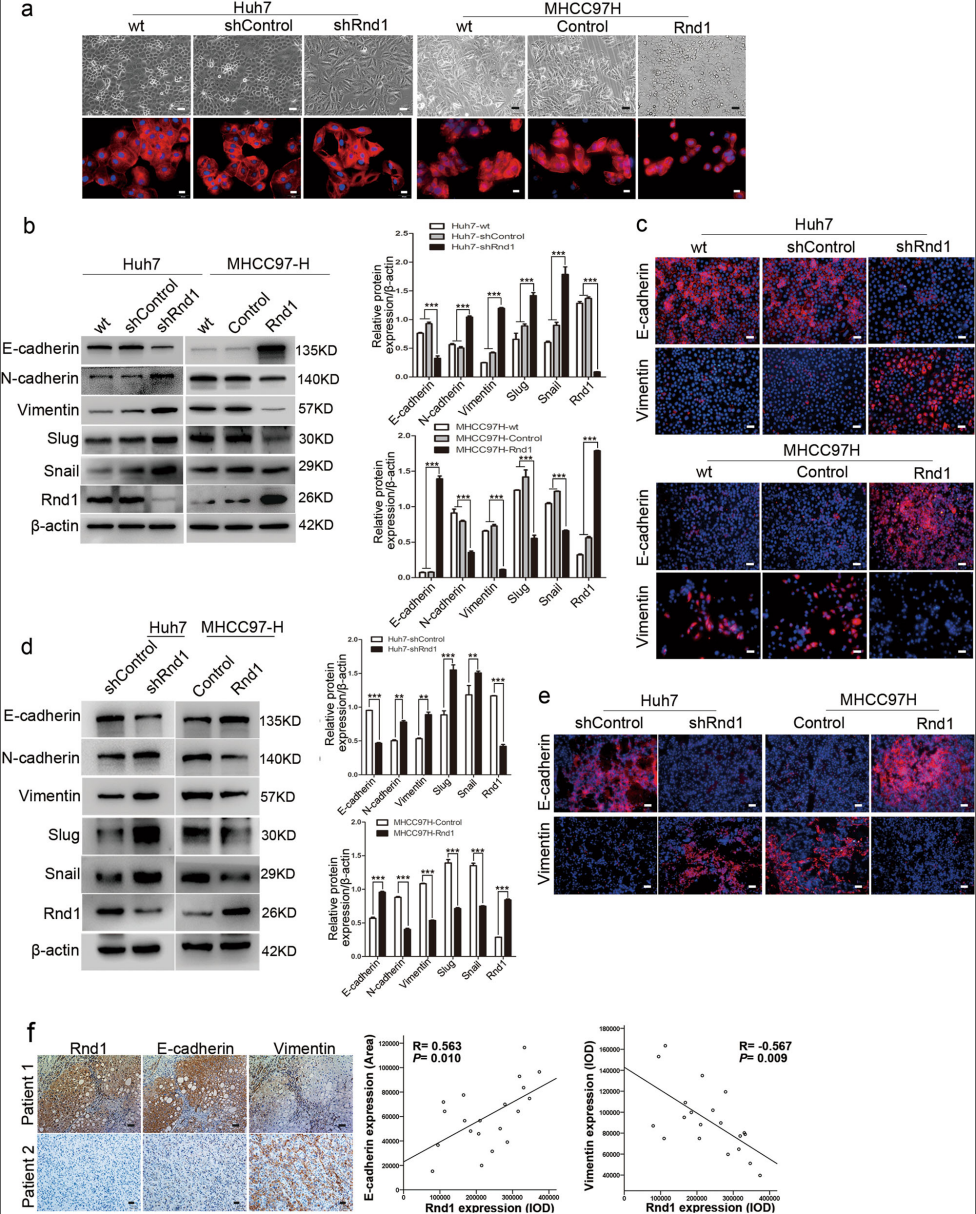
Low expression of Rnd1 induces EMT in HCC cells. **a** (upper) Representative phase-contrast images of Huh7-shRnd1 cells, MHCC97H-Rnd1 cells, and their respective wild type and control cells. **a** (lower) Representative IF images of cytoskeleton in Huh7-shRnd1 cells, MHCC97H-Rnd1 cells, and their respective wild type and control cells. Scale bar: 50 μm (upper), 20 μm (lower). **b** Western blot analysis of E-cadherin, N-cadherin, vimentin, Slug, and Snail protein expression in HCC cells with knockdown or overexpression of Rnd1 and their corresponding wild type and control cells. Densitometry of western blot in panel. Data are mean ± S.D. (*n* = 3) and are representative of three independent experiments. Significance was determined using two-way ANOVA. ***P < 0.001. **c** IF staining for E-cadherin and vimentin in Huh7, Huh7-shControl, Huh7-shRnd1, MHCC97H, MHCC97H-Control, and MHCC97H-Rnd1 cells. 4′,6-diamidino-2-phenylindole (DAPI) was used to stain nuclei. Scale bar: 50 μm. **d** Western blot analysis of E-cadherin, N-cadherin, vimentin, slug, snail, and Rnd1 expression in HCC tissues derived from orthotopic xenograft tumor models. Densitometry of western blot in panel. Data represent means ± S.D. (*n* = 3) of three independent experiments. Significance was determined using two-way ANOVA. *P < 0.05, **P < 0.01, ***P < 0.001. **e** Representative IF images of E-cadherin and vimentin from Huh7-shControl, Huh7-shRnd1, MHCC97H-Control, and MHCC97H-Rnd1 tumors in nude mice. Scale bar: 50 μm. **f** A positive association between Rnd1 and E-cadherin, however, a negative association between Rnd1 and vimentin is observed in serial slices of hepatoma tissue (*n* = 20; *R* = 0.563, *P* = 0.010 and *R* = −0.567, *P* = 0.009, respectively). Scale bar: 100 μm (upper), 20 μm (lower)

Because the Rho GTPase family is involved in modulating cytoskeletal reconstruction^9^, we therefore evaluated the impact of Rnd1 on cytoskeleton remodeling of HCC cells. Rnd1 silencing induced spindle-like mesenchymal morphology in Huh7 cells (Fig. 3a), and overexpression of Rnd1 induced a change from a mesenchymal-like shape to a cobblestone-like epithelial morphology in MHCC97H cells (Fig. 3a). As the EMT process is critical in tumor metastasis and there is a close relationship between EMT and cytoskeletal reconstruction^16,17^, the expression of epithelial and mesenchymal markers and related molecules which could lead to EMT in HCC cells were evaluated. Western blot analysis confirmed reduced epithelial marker (E-cadherin) and increased mesenchymal markers (N-cadherin, Vimentin, slug, and snail) accompanied with Rnd1 knockdown in Huh7 cells (Fig. 3b). Whereas increased Rnd1 manifested the opposite effects in MHCC97H cells (Fig. 3b). Moreover, immunofluorescence assay demonstrated that Rnd1 expression had a positive and negative effect on the fluorescence intensity of E-cadherin and vimentin, respectively (Fig. 3c).

In the orthotopic xenograft models, tissues derived from Huh7-shRnd1 tumors expressed lower E-cadherin protein levels but higher levels of mesenchymal markers compared with the control group (Fig. 3d, e). In contrast, opposite effects of E-cadherin and mesenchymal markers were observed in tissues derived from MHCC97H-Rnd1 tumors compared to controls (Fig. 3d, e). Additionally, IHC for serial slices of human tumor HCC tissue demonstrated that Rnd1 expression is positively correlated with E-cadherin (*R* = 0.563, *P* = 0.010) and negatively correlated with vimentin expression (*R* = −0.567, *P* = 0.009; Fig. 3f). These results indicated that Rnd1 might act as an inhibitor of EMT in HCC.

### Rnd1 inhibits the Raf/MEK/ERK signaling pathway in HCC

**Fig. 4:**
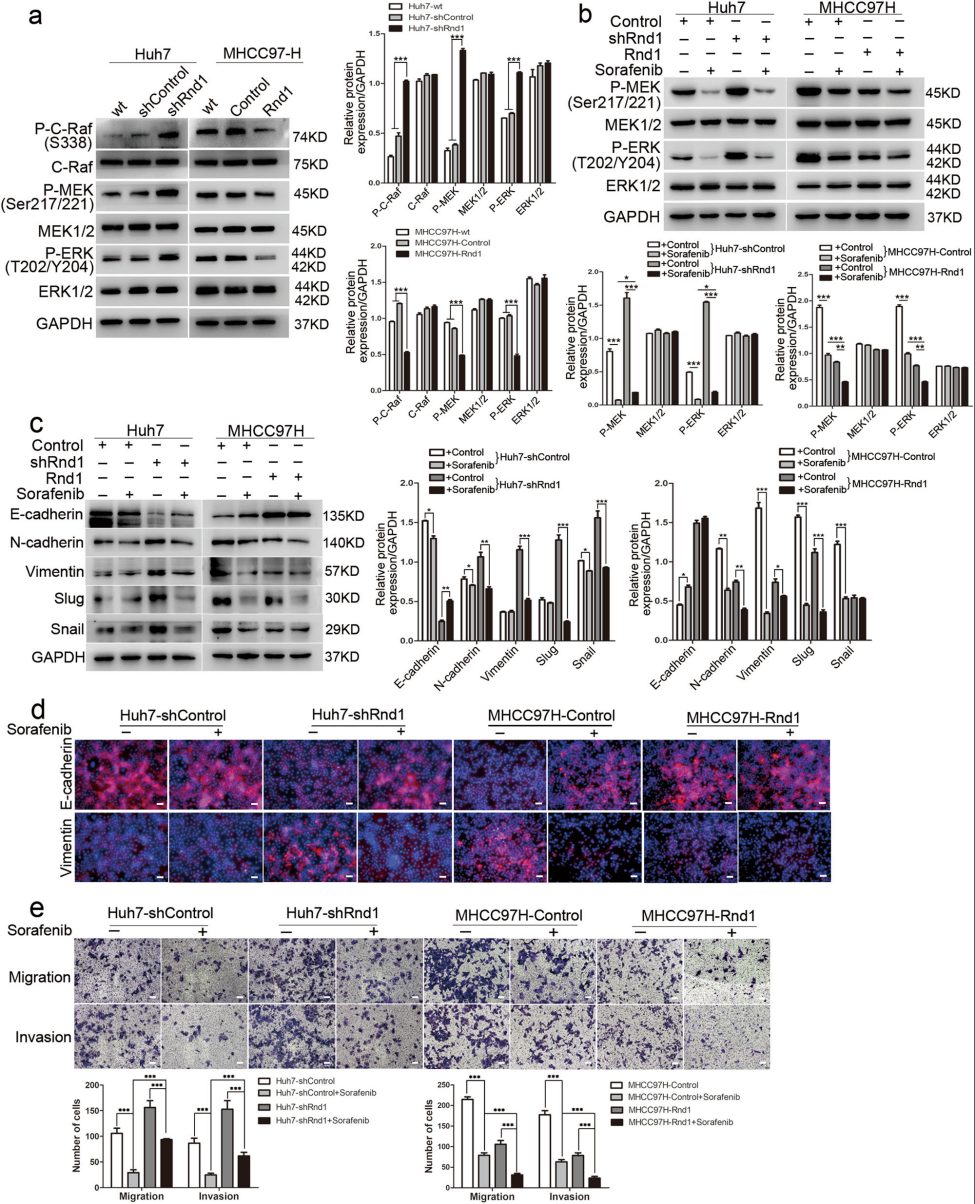
Silencing of Rnd1 induces EMT in HCC cells by the activating Raf/MEK/ERK signaling pathway. **a** Western blot analysis of the phosphorylation of Raf, MEK, and ERK in Huh7-shRnd1 cells, MHCC97H-Rnd1 cells, and their corresponding wild type and control cells. All data for the densitometric analysis of the western blots are presented as mean ± S.D. (*n* = 3) of three independent experiments. Significance was determined using two-way ANOVA. ***P < 0.001. **b** Western blot analysis of the phosphorylation levels of Raf, MEK, and ERK in Huh7-shControl/Huh7-shRnd1 and MHCC97H-Control/ MHCC97H-Rnd1 cells after treated with sorafenib for 24 h. Densitometry of western blot in panel. Data are mean ± S.D. (*n* = 3) and are representative of three independent experiments. Significance was determined using two-way ANOVA. ***P < 0.001. **c** Western blot for E-cadherin, N-cadherin, vimentin, Slug, and Snail revealed that sorafenib reverses EMT in Huh7-shRnd1 and MHCC97H-Control cells after 72 h treatment. Densitometry of western blot in panel. Data are mean ± S.D. (*n* = 3) and are representative of three independent experiments. Significance was determined using two-way ANOVA. ***P < 0.001. **d** Representative IF images of E-cadherin and vimentin in Huh7-control/+sorafenib, Huh7-shRnd1/+sorafenib, MHCC97H-control/+sorafenib, and MHCC97H-Rnd1/+sorafenib. Scale bar: 50 μm. **e** Representative images of transwell assays with or without Matrigel® for Huh7-shRnd1 cells, MHCC97H-Rnd1 cells, and their corresponding control cells with or without sorafenib treatment. Scale bar: 100 μm. Panel: quantification of migration and invasion of cells, data represent means ± S.D. (*n* = 3) of three independent experiments, significance was determined using one-way ANOVA. ***P < 0.001

It was reported that Rnd1 restricted the activity of MEK and ERK in breast cancers^14^, and hyper-activation of Raf/MEK/ERK signaling induces EMT in cancer cells^18,19^. Consistently, we found that knockdown of Rnd1 in Huh7 cells significantly increased the phosphorylation levels of Raf, MEK, and ERK (Fig. 4a), whereas ectopic expression of Rnd1 in MHCC97H cells decreased Raf, MEK, and ERK phosphorylation (Fig. 4a). To determine the necessity of Raf/MEK/ERK pathway in Rnd1-medicated functions, we blocked the Raf/MEK/ERK pathway with sorafenib, a serine/threonine kinase inhibitor that targets c-Raf and both mutant and wild-type B-Raf^20^. The phosphorylation of MEK and ERK in Huh7-shControl and MHCC97H-Rnd1 cells with higher-expression of Rnd1 was inhibited more pronounced by sorafenib than in Huh7-shRnd1 and MHCC97H-Control cells with lower expression of Rnd1 (Fig. 4b). Moreover, Sorafenib restrained the EMT-promoting effect of Rnd1 silencing in Huh7-shRnd1 and MHCC97H-Control cells (Fig. 4c, d). Transwell assays showed that sorafenib had a more pronounced effect on repressing the invasiveness and migratory ability of HCC cells with higher expression of Rnd1 than the cells with lower expression of Rnd1 (Fig. 4e). These results suggested that there was a synergism between Rnd1 and sorafenib in suppressing the activity of Raf/MEK/ERK signaling pathway, as well as the migration and invasion ability of HCC cells.

The MEK inhibitor U0126 was also used to further assess the relationship between Rnd1 and Raf/MEK/ERK pathway. Western blotting demonstrated that the high expression of p-ERK induced by Rnd1 downregulation was suppressed by U0126 (Supplementary Figure 7a). And the mesenchymal-like properties were also attenuated, as indicated by the changes of protein level and fluorescence intensity of EMT markers (Supplementary Figure 7b, c). Furthermore, Transwell assay showed that U0126 inhibited cell migration and invasion, and this effect was more pronounced in Rnd1 overexpressing cells (Supplementary Figure 7d). Collectively, these data suggested that a close interaction existed between Rnd1 and the Raf/MEK/ERK pathway.

### Rnd1 regulates the Raf/MEK/ERK pathway by modulating the activity of RhoA

**Fig. 5:**
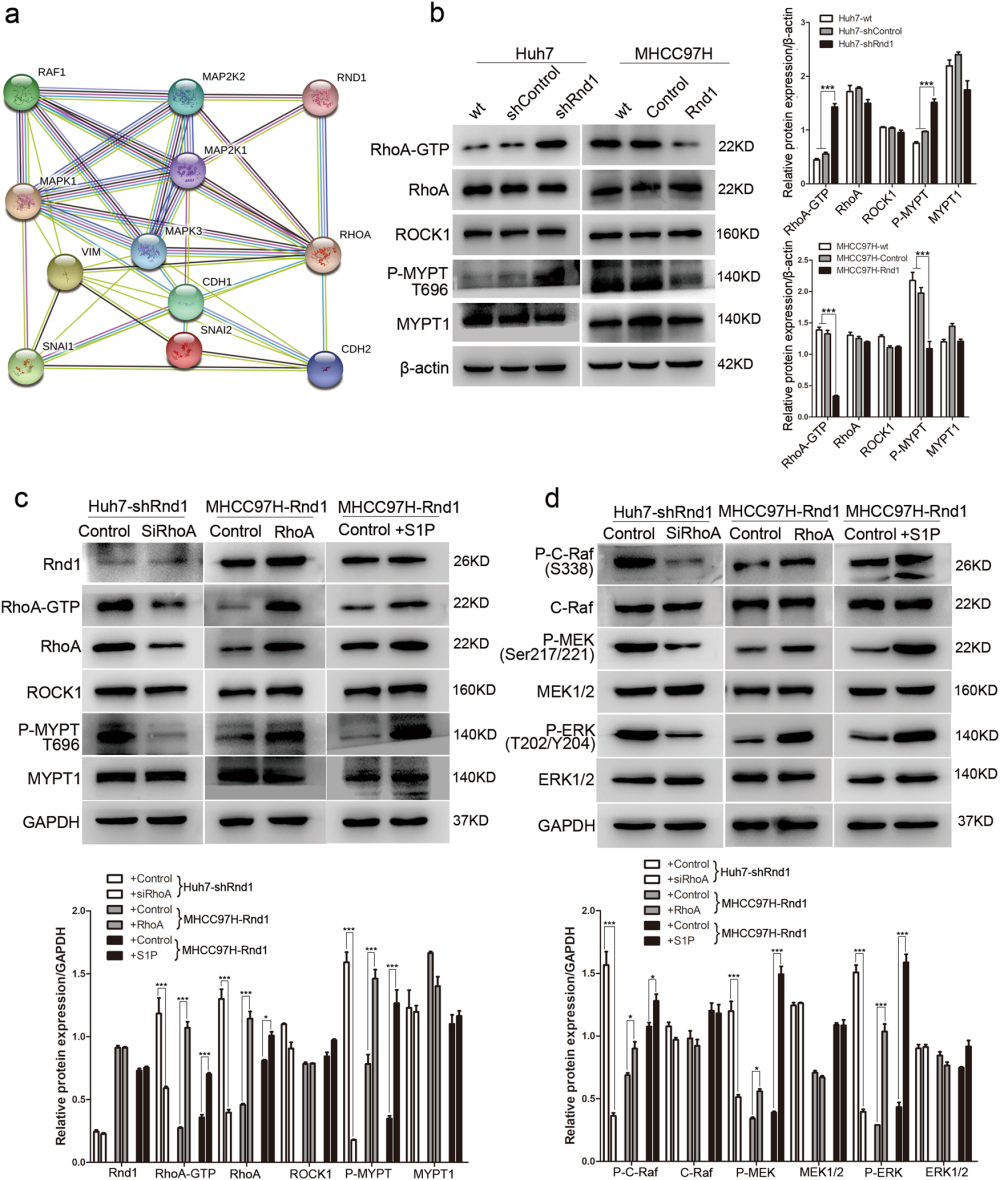
Attenuated Rnd1 expression activates Raf/MEK/ERK signaling by enhancing the activity of RhoA. **a** The interaction diagram of Rnd1 with RhoA, Raf/MEK/ERK, and EMT related molecules, as analyzed by STRING 10.0. **b** Western blot analysis of RhoA-GTP pull-down assay, indicating the degree of RhoA activation, and protein expression of ROCK1 and P-MYPT(T696), in Huh7-shRnd1 cells, MHCC97H-Rnd1 cells, and their corresponding wild type and control cells. **c** Changes in protein expression of Rnd1, RhoA-GTP, ROCK1, and P-MYPT (T696) in HCC cells with modulation of Rnd1 treated with RhoA siRNA, ectopic expression of RhoA, or stimulated with S1P. **d** Changes in protein expression of P-C-Raf (S338), P-MEK (Ser217/221), and P-ERK (T202/Y204) in HCC cells with modulation of Rnd1 treated with RhoA siRNA, ectopic expression of RhoA, or stimulated with S1P. All data for the densitometric analysis of the western blots are presented as mean ± S.D. (*n* = 3) of three independent experiments. Significance was determined using two-way ANOVA. *P < 0.01, ***P < 0.001

STRING 10.0 software was used to constructed the protein–protein interaction networks, which revealed that Rnd1 had a strong connection with RhoA (Supplementary Figure 8), a protein that plays a key role in EMT by regulating cytoskeleton organization, stress fiber bundling, and focal adhesion^21–23^. The STRING database also exhibited that RhoA could interact with the Raf/MEK/ERK signaling pathway (Fig. 5a). So we hypothesized that Rnd1 might modulate Raf/MEK/ERK signaling by interacting with RhoA. Since RhoA cycles between an active GTP-bound form and an inactive GDP-bound form^24^, therefore we examined the level of RhoA activation by using a RhoA-GTP pull-down assay. Knockdown of Rnd1 in Huh7 cells upregulated RhoA-GTP expression; in contrast, overexpression of Rnd1 in MHCC97H cells downregulated RhoA-GTP expression (Fig. 5b). ROCK1 (rho-associated, coiled-coil-containing protein kinase 1), a main downstream mediator of Rho GTPase signaling, regulates the cytoskeleton by stimulating phosphorylation of MYPT1 (myosin-associated phosphatase type 1)^25–27^, and phosphorylated MYPT1 is regarded as an indicator of ROCK1 activation^28^. The phosphorylation level of MYPT1 in Huh7-shRnd1 and MHCC97H-Control cells was higher than in the Huh7-shControl and MHCC97H-Rnd1 cells (Fig. 5b), suggesting that high expression of Rnd1 suppressed the activation of the RhoA/ROCK1/MYPT1 signaling axis.

**Fig. 6:**
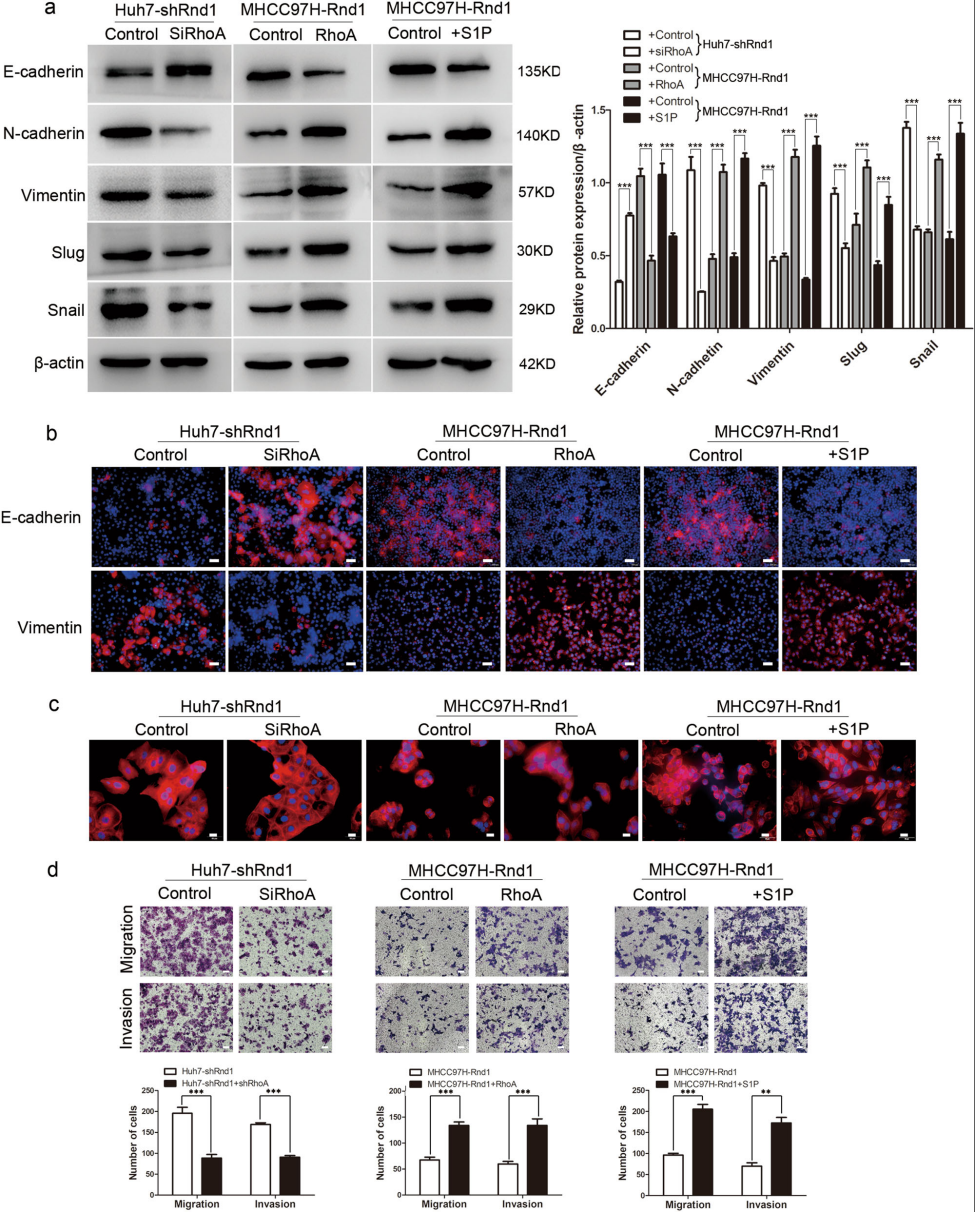
Rnd1 mediates the migration, invasion, and EMT of HCC cells by regulating RhoA activity. **a** Western blot analysis of E-cadherin, N-cadherin, vimentin, Slug, and Snail in HCC cells with modulation of Rnd1 treated with RhoA siRNA, ectopic expression of RhoA, or stimulated with S1P. Densitometry of western blot in panel. Data are mean ± S.D. (*n* = 3) and are representative of three independent experiments. Significance was determined using two-way ANOVA. ***P < 0.001. **b** Representative IF images of E-cadherin and vimentin in HCC cells with modulation of Rnd1 treated with RhoA siRNA, ectopic expression of RhoA, or stimulated with S1P. Scale bar: 50 μm. **c** The cytoskeleton of HCC cells with modulation of Rnd1 treated with RhoA siRNA, ectopic expression of RhoA, or stimulated with S1P. Scale bar: 20 μm. **d** Migration and invasion were assessed using transwell assays with or without Matrigel® in HCC cells with modulation of Rnd1 treated with RhoA siRNA, ectopic expression of RhoA, or stimulated with S1P. Scale bar: 100 μm. Panel: quantification of migration and invasion of cells. Data are mean ± S.D. (*n* = 3) and are representative of three independent experiments. Significance was determined using one-way ANOVA. ***P < 0.001

To assess the importance of RhoA in Rnd1-mediated signal pathway and functions, we modulated the expression of RhoA in HCC cells. Inhibition of RhoA by siRNA in Huh7-shRnd1 cells had no effects on the expression of Rnd1, but reduced the expression of RhoA, RhoA-GTP, and P-MYPT1 (Fig. 5c), and downregulated the phosphorylation of Raf, MEK, and ERK (Fig. 5d). Furthermore, RhoA knockdown increased E-cadherin expression and reduced the expression of mesenchymal markers in Huh7-shRnd1 cells (Fig. 6a, b). Additionally, the shape of Huh7-shRnd1-siRhoA cells changed from a spindle-like appearance to a cobblestone-like appearance (Fig. 6c). Huh7-shRnd1-siRhoA cells also exhibited reduced migration and invasive ability (Fig. 6d). These results indicated that the inhibition RhoA reversed the activation of the Raf/MEK/ERK pathway and EMT that was induced by Rnd1 silencing.

The results of rescue assays showed that ectopic expression of RhoA in MHCC97H-Rnd1 cells increased the activity of RhoA and the phosphorylation of MYPT1 (Fig. 5c), as well as the activity of Raf/MEK/ERK pathway (Fig. 5d). MHCC97H-Rnd1-RhoA cells exhibited downregulation of E-cadherin and upregulation of mesenchymal markers (Fig. 6a, b). Additionally, MHCC97H-Rnd1-RhoA cells presented a spindle-like morphology and exhibited increased migration and invasive ability (Fig. 6c, d). Treatment of MHCC97H-Rnd1 cells with the Rho-activator sphingosine-1-phosphate (S1P) dramatically induced activation of the RhoA/ROCK1/MYPT1 pathway (Fig. 5c), and resulted in the reactivation of the Raf/MEK/ERK pathway (Fig. 5d). After treatment with S1P, MHCC97H-Rnd1 cells presented a mesenchymal-like phenotype, based on cell shape and expression of key EMT molecules (Fig. 6a–c), and also exhibited augmented migratory and invasive ability (Fig. 6d). These observations suggested that the reactivation of RhoA abolished the inhibitory effect of Rnd1 on the Raf/MEK/ERK pathway and related EMT phenomena.

### Epigenetic mechanisms for the inactivation of Rnd1 in HCC

**Fig. 7:**
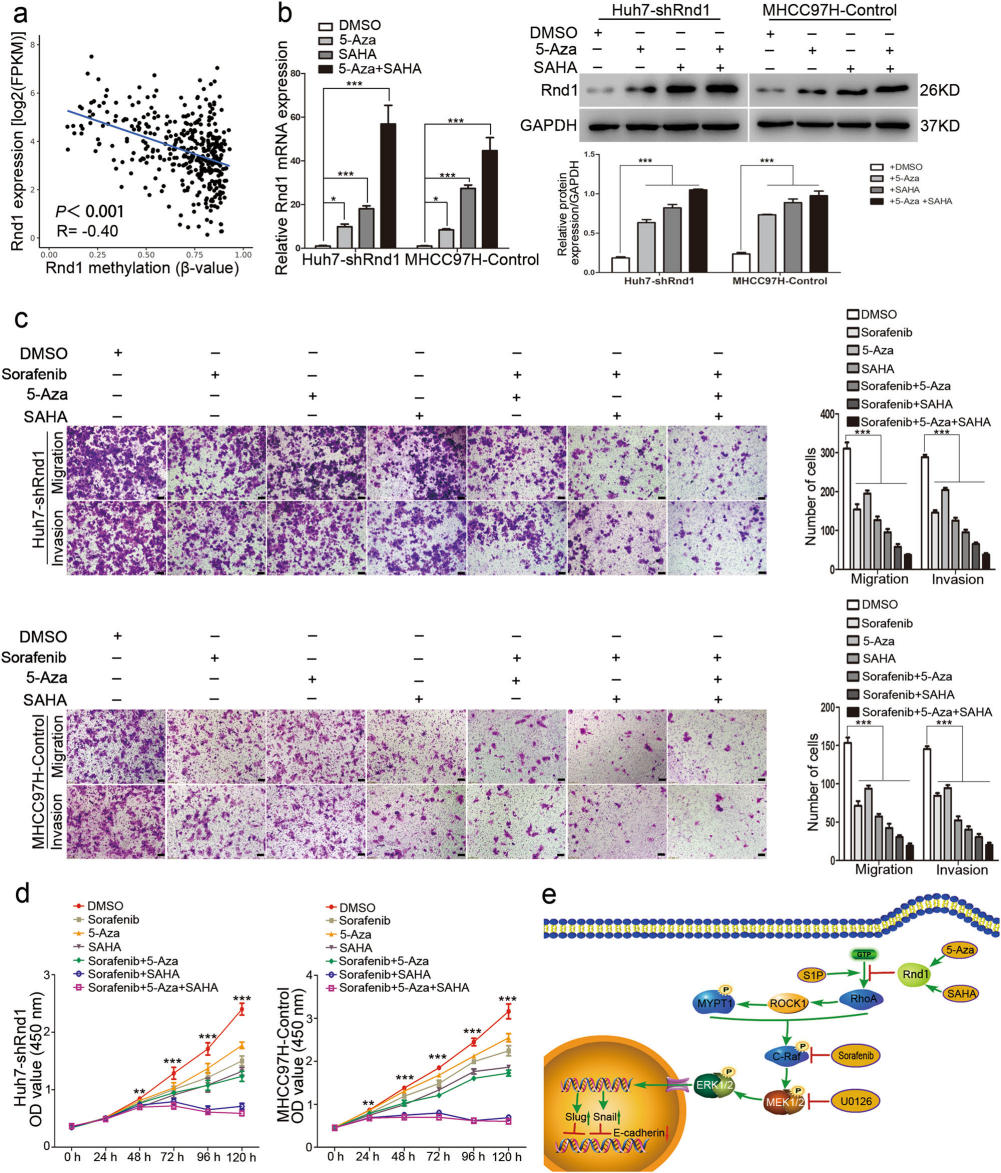
The contribution of epigenetic mechanisms to Rnd1 silencing in HCC. **a** The plot showed the relationship between Rnd1 expression and promoter methylation in TCGA data set. *n* = 396. **b** Detection of Rnd1 by Real-time PCR and Western blot in Huh7-shRnd1 and MHCC97H-Control cells after incubated with either 5-Aza (10 µM), SAHA (5 µM), or both compounds for 24 h. All data represent mean ± S.D. (*n* = 3) for three independent experiments. Significance was determined using one-way ANOVA. *P < 0.01, ***P < 0.001. **c** The cell migration and invasion evaluated by transwell assays with or without Matrigel in Huh7-shRnd1 and MHCC97H-Control cells treated with DMSO, sorafenib, 5-Aza, SAHA, sorafenib+5-Aza, sorafenib+SAHA or their mixture. Scale bar: 100 μm. Panel: quantification of migration and invasion of cells. Data are mean ± S.D. (*n* = 3) and are representative of three independent experiments. Significance was determined using one-way ANOVA. ***P < 0.001. **d** Changes in the growth rate of Huh7-shRnd1 and MHCC97H-Control cells treated with DMSO, sorafenib, 5-Aza, SAHA, sorafenib+5-Aza, sorafenib+SAHA or their mixture. Data are mean ± S.D. (*n* = 4) and are representative of three independent experiments. Significance was determined using two-way ANOVA. **P < 0.01, ***P < 0.001. **e** Schematic of Rnd1 suppressing the Raf/MEK/ERK cascade by inhibiting the activation of RhoA and restraining EMT in HCC, and Rnd1 silencing could be overcome by 5-Aza and SAHA

TCGA data sets were also employed to explore the underlying mechanisms of Rnd1 downregulation in HCC. The results revealed that Rnd1 expression was negatively correlated with promoter methylation in HCC (*R* = −0.40, *P* < 0.001; Fig. 7a), indicating that promoter methylation contributed to Rnd1 silencing in HCC. Furthermore, the results also exhibited that genetic alteration, including copy number variation and somatic mutation, had no influence on the expression of Rnd1 (Supplementary Figure 9a,b). Besides, these results were consistent with earlier research^29^. A recent study suggested that histone deacetylation was involved in the transcriptional repression of Rnd1;^14^ so the DNA methylation inhibitor 5-aza-2-deoxycytidine (5-Aza) and the histone deacetylase inhibitor SAHA were used to treat Huh7-shRnd1 and MHCC97H-Control cells with low expression of Rnd1. Western blot and Real-time PCR confirmed that 5-Aza, SAHA and both compounds could rescue Rnd1 expression in both cell lines (Fig. 7b). Next, transwell assays demonstrated that 5-Aza, SAHA, or both compounds could reinforce the inhibitory effect of sorafenib on the migration and invasion of HCC cells (Fig. 7c). CCK-8 assays also showed that the proliferating rate of Huh7-shRnd1 and MHCC97H-Control cells treated by sorafenib cooperated with 5-Aza, SAHA or both compounds was markedly slower as compared with sorafenib-treated and control cells (Fig. 7d). These results suggested that epigenetic inhibitors (5-aza and SAHA) potentiated sorafenib-induced toxicity in Rnd1-downregulated HCC cells.

Thus, we conclude that Rnd1 suppresses the activity of RhoA, leading to the inhibition of the Raf/MEK/ERK signaling pathway, and impairing the migration, invasion and EMT of HCC cells; in addition, Rnd1 silencing can be rescued by 5-Aza and SAHA in HCC cells (Fig. 7e).

## Discussion

HCC is aggressive and displays a strong tendency to both recurrence and metastasis^30^. A better understanding of the underlying mechanisms for these aspects will lead to better treatments for HCC. In this study, Rnd1 was found to be downregulated in HCC tissues and cells. Through in vitro and in vivo experiments, it was found that Rnd1 regulated EMT progression by modulating the Raf/MEK/ERK pathway through altered RhoA activity, which in turn affected cell migration and invasion. Besides, both DNA methylation and histone deacetylation were key factors for Rnd1 downregulation in HCC cells. Collectively, Rnd1 acted as a promising anti-metastasis target for HCC patients.

Owning to the involvement of Rho GTPases in many essential physiological processes of normal cells, deregulation of Rho GTPases endow cells with the hallmarks of malignancy, including malignant transformation, cancer cell survival, and metastasis^31^. In this study, we revealed that Rnd1, a member of Rho GTPases, was downregulated in HCC tissues, and negatively correlated with malignant clinic pathological features and poor prognosis of HCC patients. We also verified that Rnd1 had potent inhibitory effects on cell proliferation, migration, invasion, and metastasis in HCC. Recent studies had demonstrated that Rnd1 expression was downregulated at the initiation and progression stages of breast cancer^14^. However, the exact role of Rnd1 in other types of tumors are still unclear. Our studies further confirmed the pleiotropic roles of Rnd1 in the progression of HCC.

EMT is an essential process for cancerous cells to acquire metastatic capability, following by the changes of cell shape^32^. Rnd1 had the function of modulating cytoskeleton and cell shape^33^. In this research, Rnd1-silenced cells acquired a mesenchymal-like phenotype with the downregulation of epithelial marker and the upregulation of mesenchymal markers, as well as the acquisition of elongated spindle shape. Conversely, HCC cells with Rnd1 overexpression acquired epithelial-like phenotype with upregulation of E-cadherin. These results were also verified by expressions of Rnd1 and EMT markers in xenografted tumors and HCC tissues. Therefore, Rnd1 inactivation-induced EMT in HCC.

The signaling mechanisms underlying the inhibitory effects on EMT and HCC progression mediated by Rnd1 were also investigated. Our results showed that Rnd1-restrained HCC invasion, metastasis, and EMT through inhibiting the activity of Raf/MEK/ERK signaling. Aberrant activation of Raf/MEK/ERK pathway is implicated in malignant transformation and progression of HCC and related with poor prognosis^34^. Furthermore, mounting evidences have showed that Raf/MEK/ERK signaling facilitates tumor invasion and metastasis by modulating EMT^35,36^. Therefore, molecular therapies had been developed to interfere this pathway for the treatment of HCC. The multi-kinase inhibitor sorafenib, an orally active inhibitor of Raf kinases and receptor tyrosine kinases, is the only approved systemic agent for patients with advanced HCC. Nevertheless, a considerable number of HCC patients still suffer from disease progression during sorafenib treatment, namely sorafenib resistance. Our results demonstrated that Rnd1 overexpression and sorafenib had synergistic inhibition effect on Raf/MEK/ERK signaling. Meanwhile, Rnd1 overexpression reinforced the inhibitory effect of sorafenib on cell migration, invasion and EMT. In our previous studies, sorafenib showed pro-metastasis effect in HCC^37^, but aspirin minimized this effect by increasing HTATIP2 expression through suppression of COX2^38^. Previous experiments had shown that upregulation of COX2 was depended on the activation of MAPKs^39^. Rnd1 had the function of restraining the activity of Raf, MEK, and ERK. In this scenario, Rnd1 may act like aspirin and reduce the expression of COX2 leading to alleviate the pro-metastasis effects of sorafenib in HCC. These data suggested that exogenous-induced Rnd1 might help attenuate the sorafenib resistance in HCC and provide a novel approach for improving sorafenib efficiency.

Additionally, we also found that Rnd1 modulated the Raf/MEK/ERK signaling by connecting with RhoA. The results exhibited that Rnd1 was inversely correlated with the activity of RhoA. In addition, enhancing RhoA activity could not interfere Rnd1 expression, but could offset Rnd1-induced inhibition of cell migration, invasion, and EMT, as well as the activity of Raf/MEK/ERK pathway. Recently, Bist et al. showed that a dominant-negative form of RhoA could counteract ERK-induced NF-kB activation, but they also found that the activation of RhoA is dependent on ERK^40^. These findings suggested that the two pathways could interact with each other. According to our results, Rnd1 inhibited the metastasis and EMT of HCC by decreasing the activity of RhoA/ROCK1/MYPT1/Raf/MEK/ERK signaling axis. Thus, these findings identified a novel pathway restrained by Rnd1 for its potent malignancy suppressing activity.

Finally, TCGA analysis indicated that promoter methylation was inversely associated with Rnd1 expression in HCC. DNA methylation contributed to the tumor initiation and progression^41^. Therefore, our results indicated that promoter methylation-mediated silencing of Rnd1 might be involved in the process of tumorigenesis. Furthermore, we also found that epigenetic inhibitors elevated the expression of Rnd1 in HCC cells. Aberrant epigenetic modifications were associated with the activation of oncogenes, inactivation of tumor suppressors and acquisition of drug resistance^42^. Preclinical and clinical trials had been launched to test the anti-tumor effects of epigenetic inhibitors. However, the clinical efficacy of the inhibitors was limited due to their therapeutic targets were global, so they could not selectively efficient against solid tumors^43^. Considering that Rnd1 is frequently silenced in HCC, generating specific inhibitors for Rnd1 methylation may achieve stronger tumor-selective toxicity than traditional drugs.

In a summary, our study shows that Rnd1 is negatively associated with the aggressive characteristics and poor prognosis of HCC patients. Besides, Rnd1 upregulation suppresses the activity of the Raf/MEK/ERK pathway by inhibiting RhoA activation, restraining migration, invasion, and EMT of HCC cells. The combination of pharmaceutical upregulation of Rnd1 with conventional measures may serve as a new weapon for HCC treatment in the clinical application.

## Materials and methods

### Patients and tissue specimens

We collected 67 pairs of liver cancer (T) and adjacent non-tumor tissue (NT) from treatment-naive patients who underwent hepatectomy for HCC between May 1999 and November 2002 at the Liver Cancer Institute and Zhongshan Hospital (Fudan University, Shanghai, China). The research was approved by the research ethics committee of Zhongshan Hospital. Fresh HCC tissues were snap-frozen in liquid nitrogen and stored at −80 °C for construction of tissue microarray and immunohistochemistry (IHC). The clinical and pathological features of these patients are described in Table 1. The uniform guidelines for post-operative follow-up procedures were described previously^44^.

We obtained 20 pairs of fresh HCC tissue and matched non-tumor liver tissue from patients who underwent surgical treatment for HCC between 2014 and 2016; these tissues were used for quantitative real-time polymerase chain reaction (qRT-PCR) analysis. Additionally, eight pairs of tumor/non-tumor liver tissue samples from HCC patients who underwent operative treatment in 2016 were randomly selected for western blot analyses. All human materials were obtained with informed consent.

### Cell lines

The human HCC cell lines, including HepG2, Huh7, and a normal liver cell line (L02) were purchased from the cell bank of the Chinese Academy of Sciences (Shanghai, China). HCCLM3, MHCC97H, and MHCC97L cell lines were established at the Liver Cancer Institute, Fudan University (Shanghai, China)^45^. All cell lines were cultured in Dulbecco’s modified Eagle’s medium (DMEM; Invitrogen, Carlsbad, CA) containing 10% fetal bovine serum (FBS; Gibco, Grand Island, NY) and 100 U ml^−1^ penicillin and 50 mg ml^−1^ streptomycin, and incubated at 37 °C and 5% CO_2_ in a humidified atmosphere.

### Lentiviral vector construction

Lentiviral-mediated Rnd1 pLKO/shRNAs were purchased from Genesent (Shanghai, China). The sequences of the three Rnd1-targeting lentiviral shRNA constructs were: shRNA1: 5′-GCTCTGAACTCATCTCTTCTA -3′; shRNA2: 5′-GAGGACAGAAATCCTAGATTA-3′; shRNA3: 5′-CCCTACTACGATAATGTCCGT-3′; shRNA4: 5′-GGAGCTTAGTCTCTGGGATAC-3′; shRNA5: 5′- GGAAACCTCATCTTTGCATAG -3′; shRNA6: 5′- GCGACTCGGATGCAGTATTAC -3′. The plasmid encoding Rnd1 (NM_014470.3) was synthesized by using pLenO-DCE-Puro Vector (biolink, Shanghai, China). Lenti-Rnd1 viral particles were produced by cotransfection of 293T cells with four plasmids: the pRSV-Rev packaging helper plasmid, pMDLg/pRRE, pMD2.G, and the pLenO-DCE Transfer Vector. Stably transfected clones were validated using qRT-PCR and western blotting.

### Plasmid, siRNA construction and transfection

Full-length human RhoA (NM_ 001664.2) was cloned into the control plasmid DNA vector (pcDNA 3.1) and transfected into HCC cells using Lipofectamine 2000 reagent (Invitrogen, Carlsbad, CA), according to the manufacturer’s instructions. Cells transfected with empty vector were used as controls. RhoA siRNA was purchased from Biotrend Company (Shanghai, China); the RhoA siRNA sequence was 5′-CCACAGUGUUUGAGAACUA-3′^46^. siRNA transfection of HCC cells was accomplished using Lipofectamine 2000. Cells transfected with non-targeting siRNA were used as controls.

### Active RhoA pull-down assay

GTP loading of RhoA was examined using a RhoA Activation Assay Kit (NewEast Biosciences, USA), according to the manufacturer’s protocol. Briefly, when the density of HCC cells reached about 80–90% confluence, 1 ml ice-cold 1× Assay/Lysis Buffer was added to the culture plate and collected into assay tubes. Then, 1 μl of anti-active RhoA monoclonal antibody was added into assay tubes. Afterwards, 20 µl of resuspended protein A/G Agarose beads was added to each tube, and the cell lysate mixtures were gently agitated for 1 h at 4 °C. The beads were collected by centrifugation for 1 min at 5000 × *g*, and were washed three times with 0.5 ml 1× Assay/Lysis buffer. The bead pellet was resuspended in 20 µl of 2× reducing SDS-PAGE sample buffer, and the samples were boiled for 5 min. Equivalent amounts of samples were separated by SDS-PAGE on a 12% gel and transferred to nitrocellulose membranes. RhoA expression was queried using the anti-RhoA antibody provided by the kit.

### Xenograft model of human HCC in nude mice

The male BALB/c nude mice were purchased from Shanghai SLAC Laboratory Animal Co. Ltd. (Shanghai, China) and housed under specific pathogen-free conditions. For the xenograft model, Huh7-shControl, Huh7-shRnd1, MHCC97H-Control and MHCC97H-Rnd1 cells (5 × 10^6^ cells) were injected subcutaneously into the left upper flank regions of three nude mice (4 weeks of age), respectively. After 1 month, subcutaneous tumor tissues were resected and cut into 1 mm^3^ pieces, the tumor pieces were implanted into the livers of nude mice (*n* = 6 for each group) according to standard procedures described previously^47^. After 40 days, mice were killed and tumor tissues and lungs were harvested, photographed, and weighed. The tumor volume was calculated by the following formula: tumor volume (mm^3^) = = (longest diameter × shortest diameter^2^)/2, and the incidence of lung metastasis of each group was quantified by fluorescence microscopy and HE straining. All procedures strictly followed the guidelines established by the Shanghai Medical Experimental Animal Care Commission.

### Cell migration assays, Matrigel® invasion assays and Scratch-healing assays

The Transwell Permeable Supports System (Corning) was employed to detect the effects of Rnd1 on cell migration and invasion. For assays of migration, 5 × 10^4^ cells in 1% serum medium were added to the upper chamber; for assays of invasion, 1 × 10^5^ cells in 1% serum medium were added to the Matrigel-coated upper chamber and 10% serum medium was added to the lower chamber. Cells were permitted to translocate for 24 or 48 h at 37 °C. After removal of the non-migrated or non-invaded cells, the remaining cells were fixed with methanol, stained with crystal violet. Three random views of each chamber were photographed by inverted microscopy (magnification: 100×), and the cell numbers were counted by Image-Pro Plus software. For the scratch-healing assay, Rnd1-modified HCC cells were added to 6-well plates and starved for 24 h in non-serum medium. After that, a pipette tip was used to make a straight scratch on the monolayer of cells. The scratch was randomly photographed under an inverted microscope at 0 and 48 h. The following formulas were used to quantify the results: Total scratched area (0 h) = scratch width (mm) × length (mm); cell migrated area (48 h) = length of cell migration (mm) × 2 × Length (mm); percent wound closure (%) = cell migrated area/total scratched area. All samples were prepared in triplicate on three independent experiments.

### Colony formation assays, cell proliferation assays, and cell cycle analysis

Colony formation assays were carried out in accordance with previous experiments^47^. For cell proliferation assays, 3000 cells/well were seeded in 96-well plates, and cell proliferation was measured using the Cell Counting Kit-8 (CCK-8; Dojindo Laboratories, Kumamoto, Japan), reading absorbance at 450 nm according to the manufacturer’s instructions. For the cell cycle analysis, collected HCC cells were fixed in 70% ice-cold ethanol for 12 h; after that, HCC cells were strained with PI by using PI/RNase Staining Buffer (BD Biosciences, San Jose, CA) according to the manufacturer’s protocol. Results collection and analysis were performed with a flow cytometer (FACS Calibur; BD Biosciences, San Jose, CA). Experiments were repeated three times.

### Immunofluorescence assays and TUNEL assays

HCC cells were seeded in glass bottom dishes. After cells attached to the bottom of the dishes, cells were fixed in 4% paraformaldehyde and permeabilized with 0.2% Triton X-100. Afterwards, the cells were incubated with blocking buffer (5% BSA in PBS, pH 7.4) for 30 min. Cells were incubated with the appropriate primary antibodies overnight at 4 °C. On the following day cells were incubated with Alexa Fluor 555-labeled secondary antibodies (Beyotime, Haimen, China) for 1 h and DAPI was used to mark nuclei. For the TUNEL assays, after cells were incubated with blocking buffer, the TUNEL reaction mixture (Roche, Pleasanton, CA, USA) was used to treat cells for 1 h at 37 °C in the dark place. Subsequently, cells were strained with DAPI for nuclei visualization. Apoptotic cells appeared as red-stained cells. The cells were observed and photographed under a fluorescence microscope (Olympus, Tokyo, Japan). Data are shown from a typical experiment performed in triplicate.

### Real-time PCR assay

Total RNA was extracted with TRIzol Reagent (Sigma, St. Louis, MO) and reverse-transcribed into cDNA using the PrimeScript RT reagent Kit (TaKaRa, Dalian, China), according to manufacturer’s instructions. Quantitative PCR was performed to quantify the mRNA expression of Rnd1 and GAPDH with the RT-PCR kit (TaKaRa, Dalian, China). Each sample was tested in triplicate in three independent experiments.

### Western blot assay

Total protein was extracted in RIPA lysis buffer (Beyotime, Haimen, China), as we have previously described^7^. A total of 30 µg protein from whole cell extracts was subjected to SDS-PAGE and transferred to PVDF membrane (Millipore, Bedford, MA). Membranes were blocked with 5% nonfat milk for 1 h and then incubated with the appropriate primary antibodies overnight at 4 °C. The next day, membranes were incubated with the appropriate HRP-conjugated secondary antibodies for 1 h at room temperature. Blots were visualized with an ECL detection kit (Millipore, Bedford, MA) and analyzed using Image-Pro Plus 6.0 software (Media Cybernetics, Silver Spring, USA).

### Immunohistochemistry

Formalin-fixed paraffin sections or tissue arrays containing tumor tissues from HCC patients were stained for Rnd1 using the streptavidin–peroxidase system (ORIGENE, Beijing, China). The IOD of positive staining was analyzed using Image-Pro Plus 6.0 software.

### Statistical analysis

Data were expressed as mean ± S.D.S derived from at least three independent experiments. Data were analyzed using SPSS 18.0 (Chicago, IL USA). The Student’s *t*-test was used to analyze differences between two groups, and two-way ANOVA was used when more than two groups were compared. For the in vivo assay, the differences of tumor volumes were compared by unpaired *t*-test, and *χ*^2^ test was used to analyze the differences in the incidence of lung metastasis between groups. The correlations between Rnd1 and the clinical and pathological features were analyzed using the Spearman correlation test. OS and DFS curves were protracted using the Kaplan–Meier method and estimated by the log-rank test. Variables that were independently associated with OS and DFS were identified using the Cox’s proportional hazards regression model. A two-tailed value of *P* < 0 .05 was considered significant.

## Electronic supplementary material

Supplementary Figures and legends

Supplementary table 1
