# Supplementary material for: The Rho GTPase Rnd1 inhibits epithelial–mesenchymal transition in hepatocellular carcinoma and is a favorable anti-metastasis target
Source: Cell Death Dis. 2018 Apr 30;9(5):486. doi: 10.1038/s41419-018-0517-x (PMC5924761; doi:10.1038/s41419-018-0517-x)
Supplement: Supplementary file 1 — Supplementary Figures and legends [file 41419_2018_517_MOESM1_ESM.docx]

**The Rho GTPase** **Rnd1 inhibits epithelial-mesenchymal transition in hepatocellular carcinoma and is a favorable anti-metastasis target**

Cheng-Dong Qin^2,7^, De-Ning Ma^3,7^, Shi-Zhe Zhang^1,7^, Ning Zhang^5^ , Zheng-Gang Ren^1^, Xiao-Dong Zhu^1^, Qing-An Jia^4^, Zong-Tao Chai^6^, Cheng-Hao Wang^5^, Hui-Chuan Sun^1^, Zhao-You Tang^1*^

**
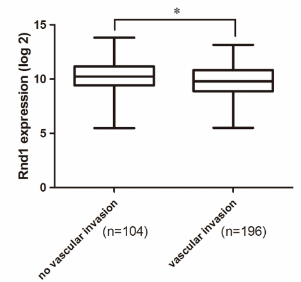
**

**Figure S1. Box plot of Rnd1 expression in HCC tissues with or without vascular invasion from the TCGA dataset.**


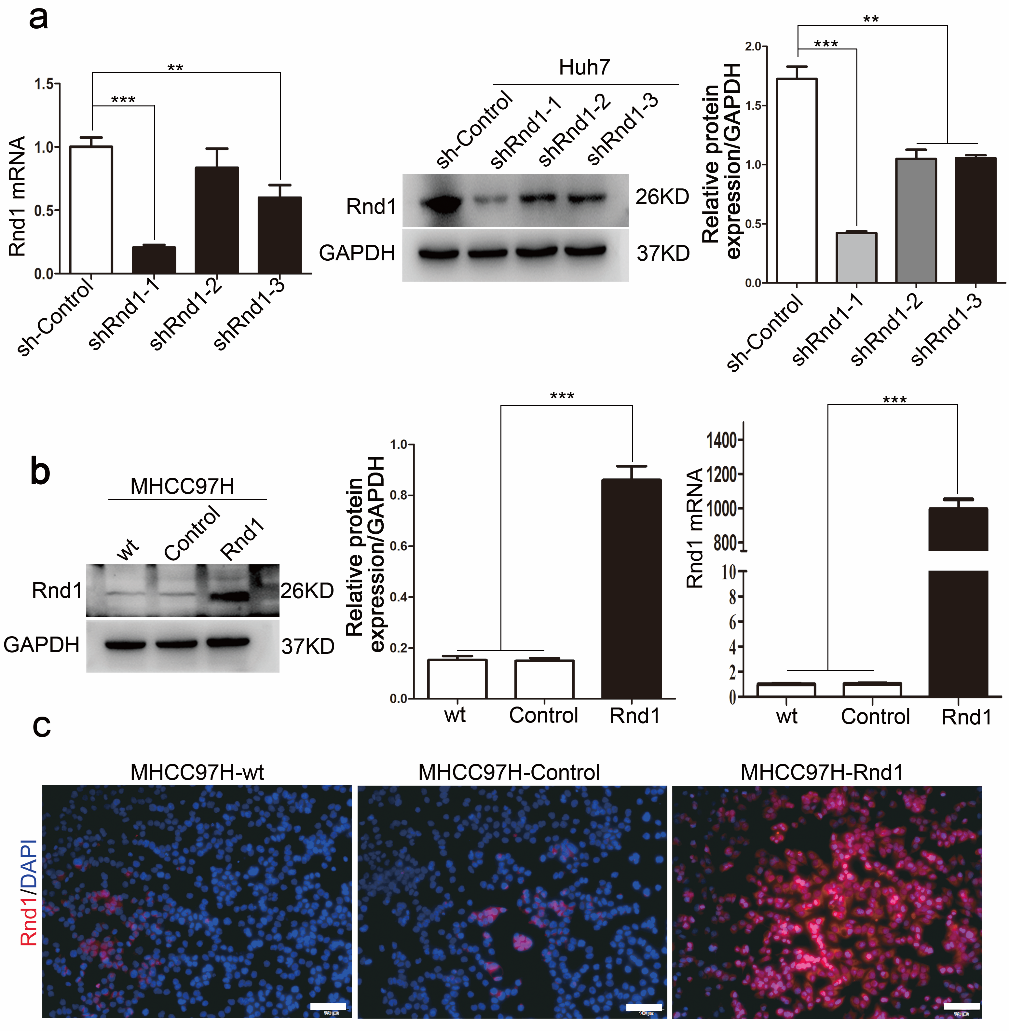


**Figure S2.** **Western blot, Immunofluorescence assay and Real-time PCR were used to evaluate Rnd1 expression in Huh7 and MHCC97H cells transfected with Rnd1 shRNA or Rnd1 cDNA.** (a) The protein and mRNA expression levels of Rnd1 in Huh7-shRnd1-1, -2, -3 cells and corresponding control cells. Panels represent mean ± S.D. (n=3) from three independent experiments plated in triplicate. Significance was determined using one-way ANOVA. ***P*＜0.01, ****P*＜0.001. (b) The protein and mRNA expression levels of Rnd1 in MHCC97H-Rnd1 cells and corresponding control cells. Data are mean ± S.D. (n=3) and are representative of three independent experiments. Significance was determined using one-way ANOVA. ***P*＜0.01, ****P*＜0.001. (c) Representative IF images of Rnd1 in MHCC97H-Rnd1 and corresponding control cells. Scale bar: 50 μm.

**
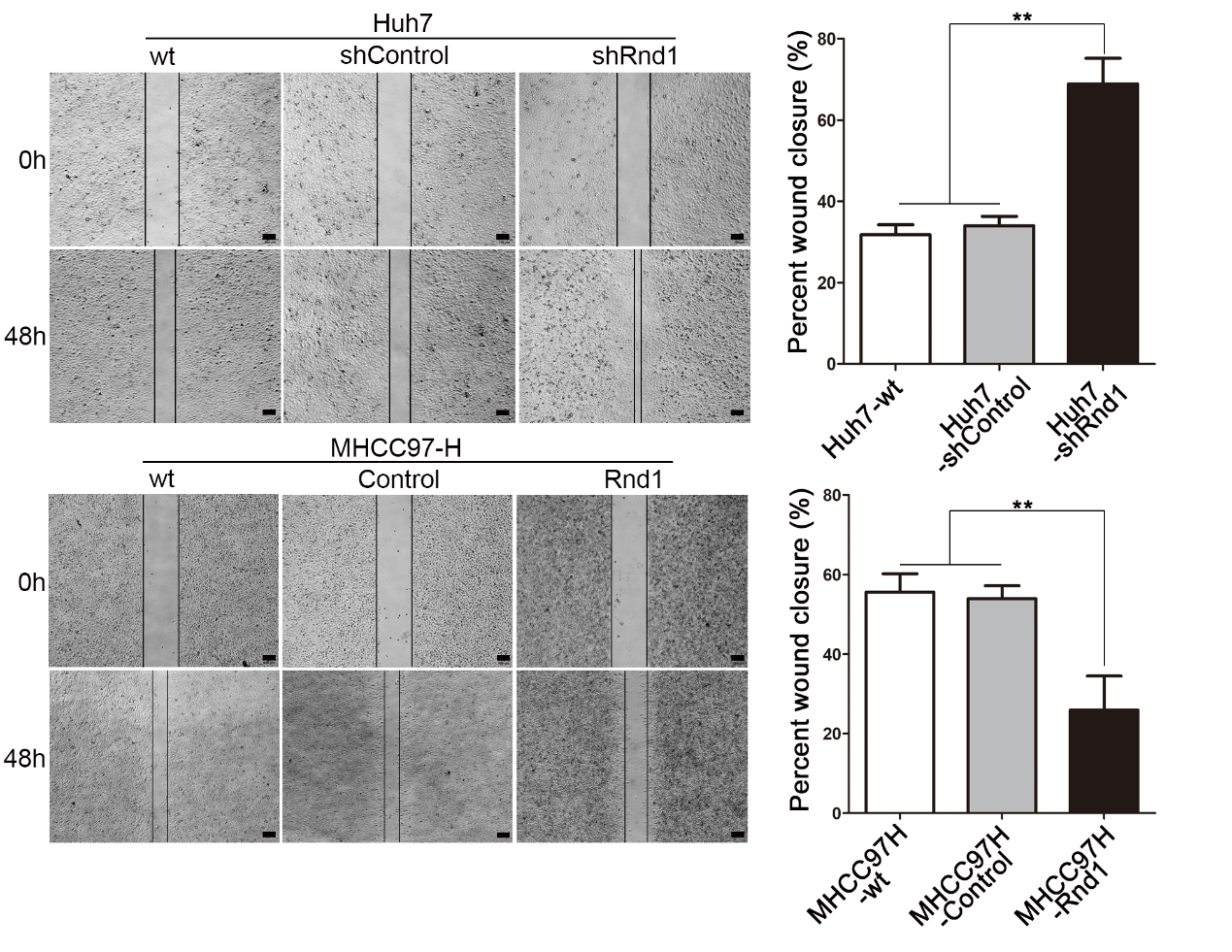
**

**Figure S3.** **Attenuated Rnd1 expression promotes the migration of HCC cells in vitro.** Effects of Rnd1 knockdown and over-expression on migration of HCC cells was evaluated in a scratch-healing assay. Representative images of 0 and 48 h time points are shown. Scale bar: 200 μm. The percent of wound closure is determined and compared in the diagrams, which are expressed as the mean ± S.D. (n=3) of three independent experiments, significance was determined using one-way ANOVA. ****P*＜0.001, ***P*＜0.01.


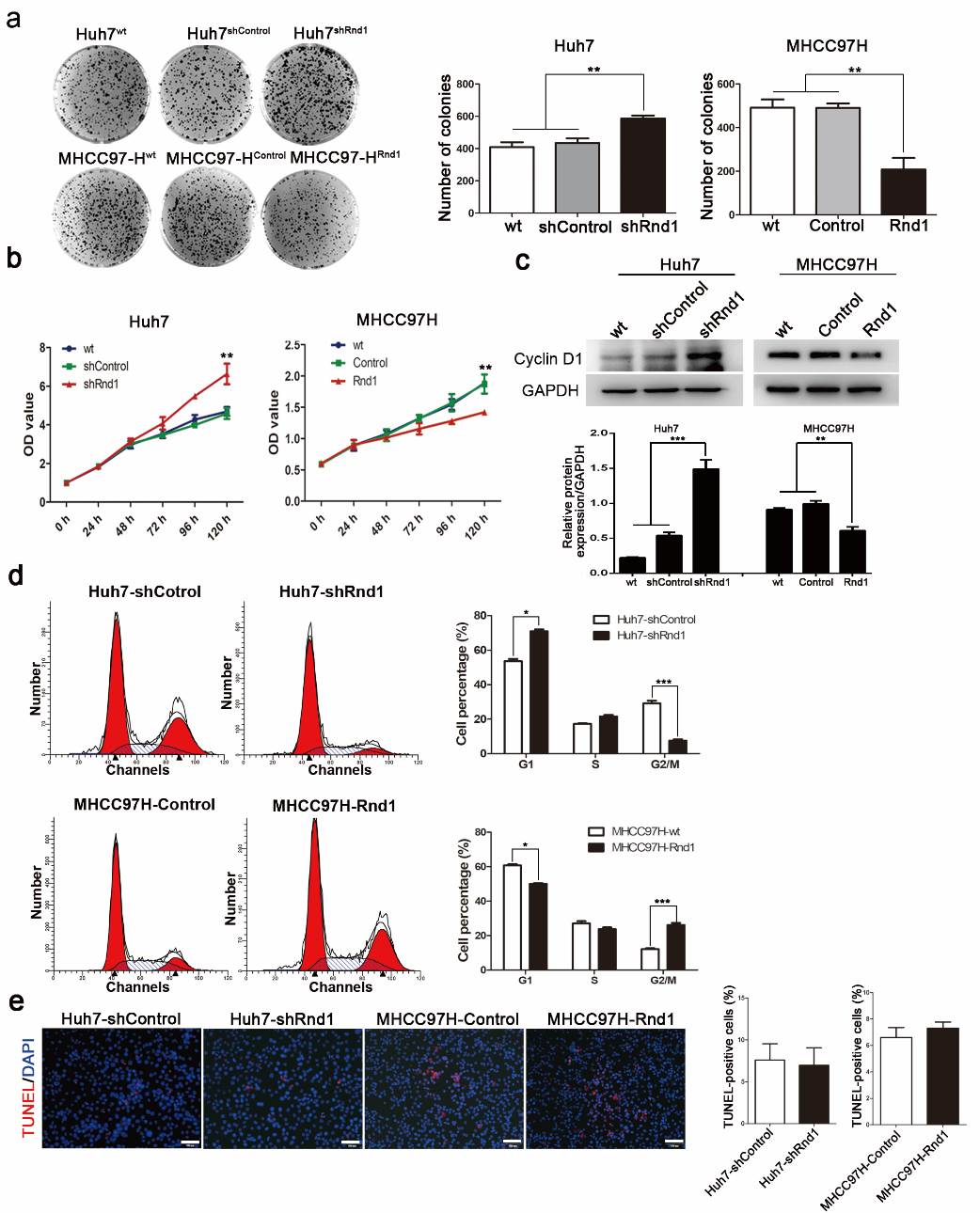


**Figure S4. Rnd1 modulated cell proliferation and cell cycle in HCC cells.** (a) Representative images of colony formation assays; the number of colonies was counted in triplicate plate and compared in the diagrams, significance was determined using one-way ANOVA. ***P*＜0.01.(b) CCK8 assay revealed that the growth rate of Huh7-shRnd1 cells and MHCC97H-Control cells were faster than the Huh7-shcontrol and MHCC97H-Rnd1 cells, respectively, all values are expressed as mean ± S.D. (n=4) of three independent experiments, significance was determined using two-way ANOVA. ***P*＜0.01. (c) Western blot analysis indicated that Rnd1-knockdown increases the protein expression of Cyclin D1 in Huh7 cells, while Rnd1-overexpression had the opposite effect in MHCC97H cells. Densitometry of western blot in panel. All values are expressed as mean ± S.D. (n=3) of three independent experiments, significance was determined using one-way ANOVA. ***P*＜0.01, *** *P*＜0.001. (d) The effect of Rnd1 on cell cycle was determined by FACS analysis. Data are mean ± S.D. (n=3) and are representative of three independent experiments, significant difference was tested by student’s t-test. **P*＜0.05, ****P*＜0.001. (e) TUNEL assays showed that Rnd1 had no effect on cell apoptosis in HCC cells. Scale bar: 50 μm. Data are presented as mean ± S.D. (n=3) of three independent experiments, significant difference was tested by student’s t-test.


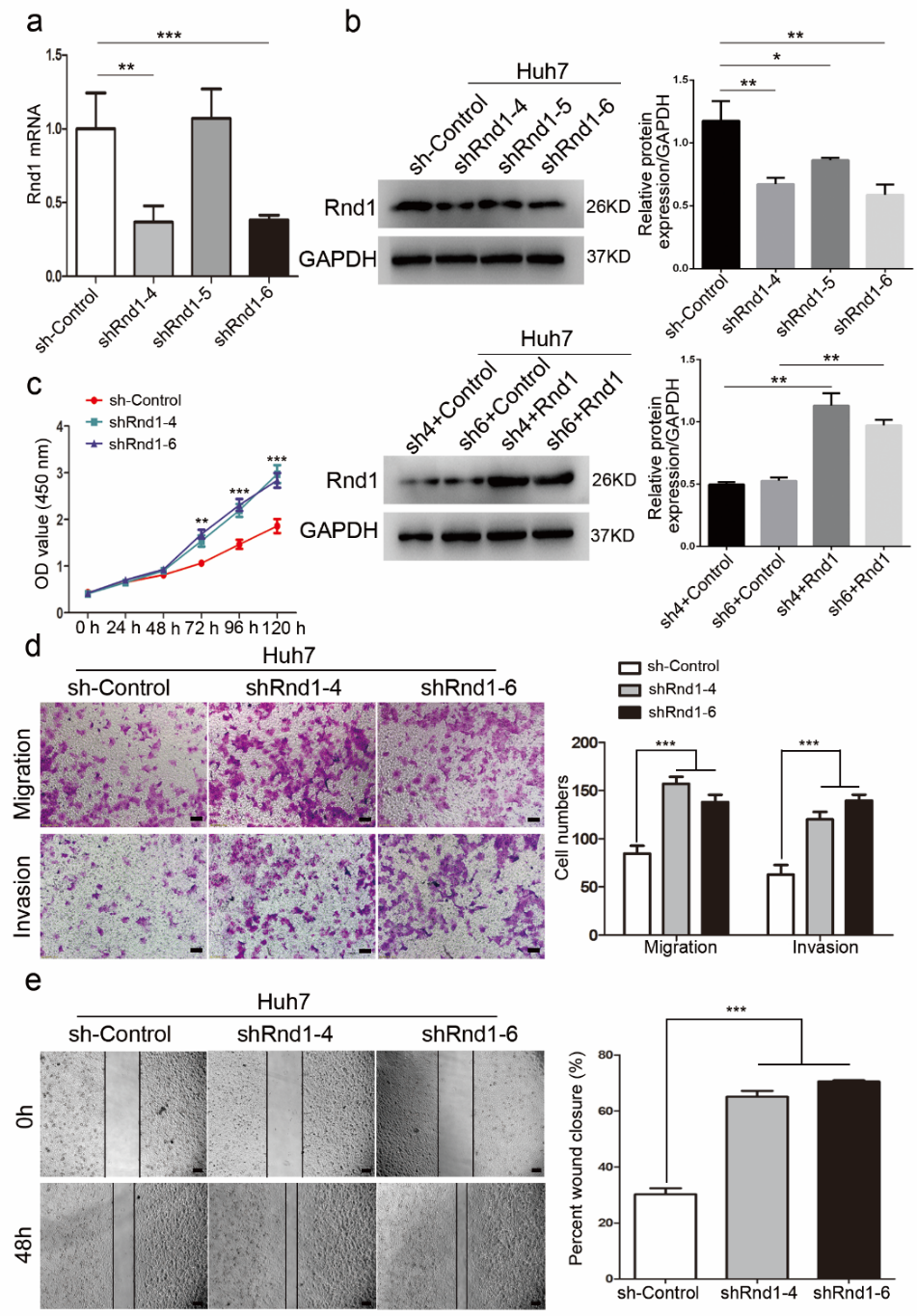


**Figure S5. Another three shRNAs were used to eliminate the possibility of off-target effects.** (a，b) The protein and mRNA expression levels of Rnd1 in Huh7-shRnd1-4, -5, -6 cells and corresponding control cells. In addition, Rnd1 cDNA was employed to recover Rnd1 expression in Huh7-shRnd1-4 and -6 cells. Densitometry of western blot in panel. Data are mean ± S.D. (n=3) and are representative of three independent experiments. Significance was determined using one-way ANOVA. ***P*＜0.01, *** *P*＜0.001. (c) CCK8 analysis of Huh7 cells transfected with shRnd1-4, -6 and control plasmid. Data represent means ± S.D. (n=4) of three independent experiments, significance was determined using two-way ANOVA. ***P*＜0.01, *** *P*＜0.001. (d) Representative images of transwell assays with or without Matrigel® for Huh7-shRnd1-4 cells, Huh7-shRnd1-6 cells and their corresponding control cells. Scale bar: 100 μm. Data are mean ± S.D. (n=3) and are representative of three independent experiments. Significance was determined using one-way ANOVA. ****P* < 0.001. (e) Effects of shRnd1-4 and -6 on migration of Huh7 cells was evaluated in a scratch-healing assay. Representative images of 0 and 48 h time points are shown. Scale bar: 200 μm. The percent of wound closure is determined and compared in the diagrams, data represent means ± S.D. (n=3) of three independent experiments, significance was determined using one-way ANOVA. ****P* < 0.001.

**
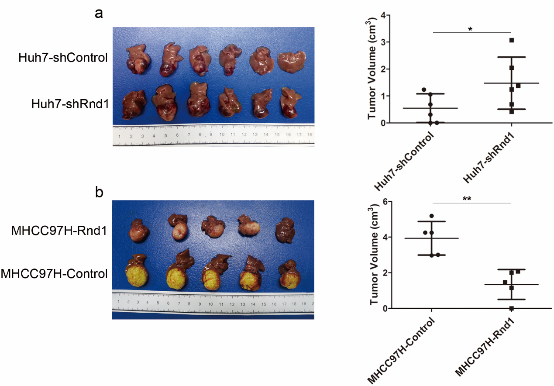
**

**Figure S6. Low Rnd1 expression promotes HCC growth *in vivo*.** (a, b) Orthotopic xenograft tumor models were constructed using Huh7-shControl (n=6) and Huh7-shRnd1 cells ( n=6; upper), or using MHCC97H-Control (n=5) and MHCC97H-Rnd1 cells (n=5; lower). The volume of tumors in each group is calculated with the formula: (Length × Width^2^)/2 and compared in the diagrams, significant difference was tested by student’s t-test. ***P*＜0.01.


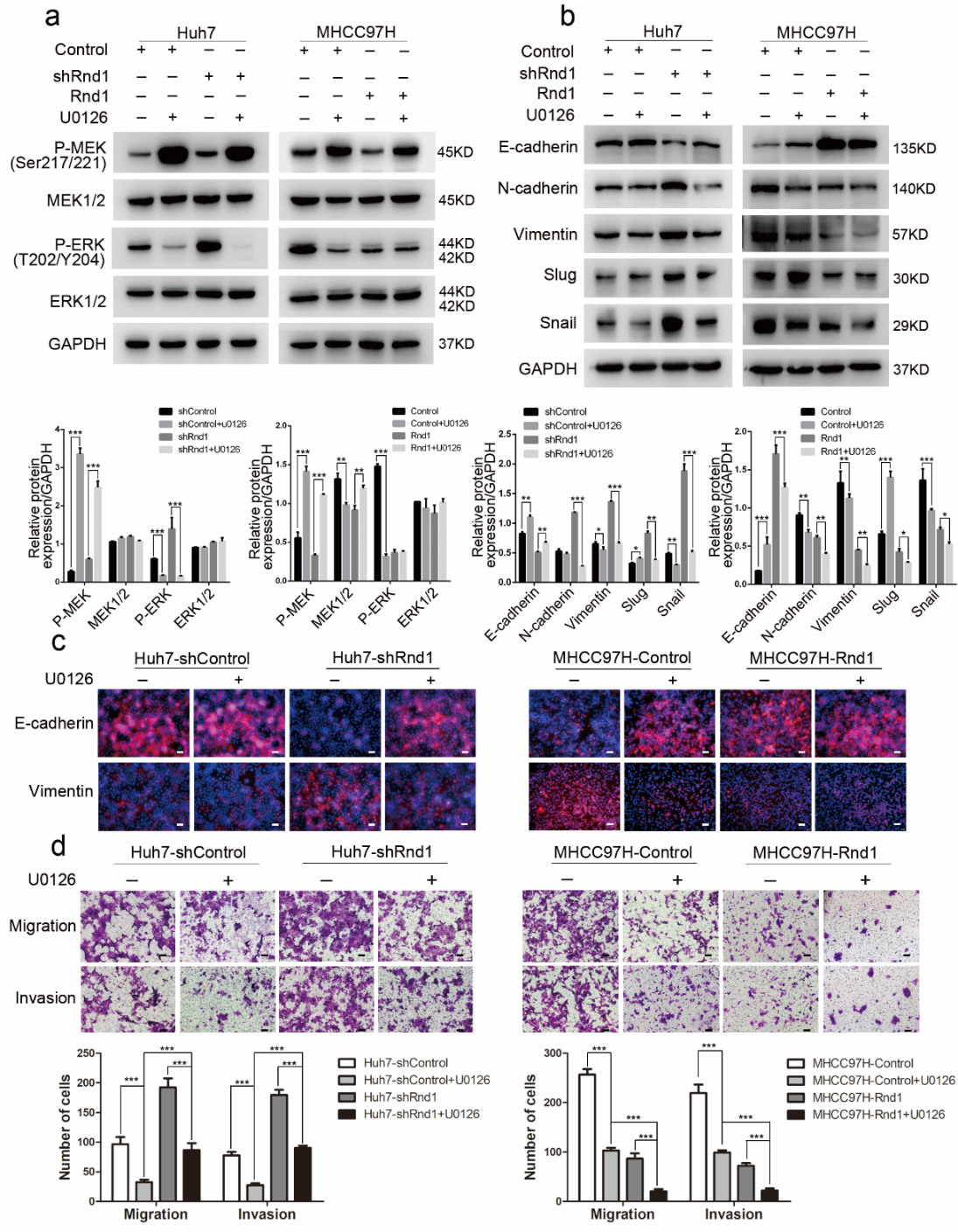


**Figure S7.** **Silencing of Rnd1 induces HCC EMT by activating the Raf/MEK/ERK signaling pathway.** (a) Changes in phosphorylation of MEK and ERK in Huh7-shControl/Huh7-shRnd1 and in MHCC97H-Control/MHCC97H-Rnd1 cells after 24 h U0126 treatment. (b) The expression changes of classical EMT markers in Huh7-shControl/Huh7-shRnd1 and in MHCC97H-Control/MHCC97H-Rnd1 cells after 24 h U0126 treatment. All data for the densitometric analysis of the western blots are presented as mean ± S.D. (n=3) of three independent experiments. Significance was determined using two-way ANOVA. **P*＜0.05, ***P*＜0.01, ****P*＜0.001. (c) Representative IF images of E-cadherin and vimentin in Huh7-control/+U0126, Huh7-shRnd1/+U0126, MHCC97H-control/+U0126, and MHCC97H-Rnd1/+U0126. Scale bar: 50 μm. (d) Representative images of transwell assays with or without Matrigel® for Huh7-shRnd1 cells, MHCC97H-Rnd1 cells, and their corresponding control cells with or without U0126 incubation. Scale bar: 100 μm. Data are mean ± S.D. (n=3) and are representative of three independent experiments. Significance was determined using one-way ANOVA. ****P* < 0.001.

**
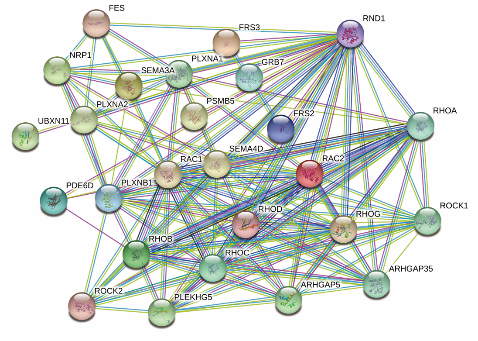
**

**Figure S8. The interaction diagram of correlated molecules with Rnd1 analyzed by STRING 10.0.**


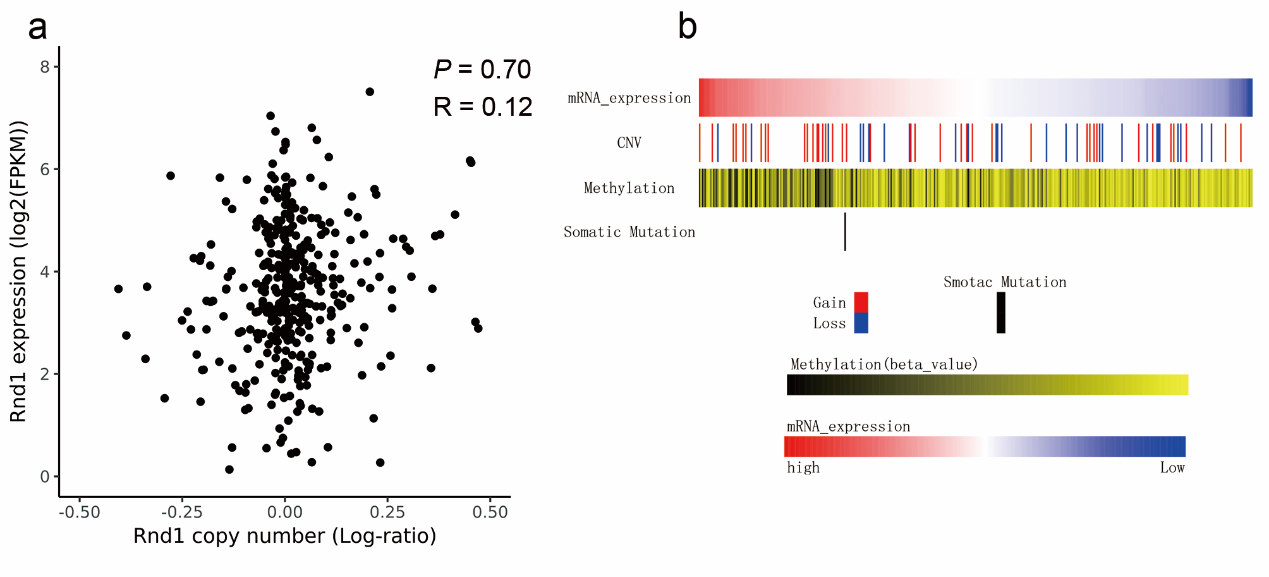


**Figure S9. Correlation of Rnd1 expression to** **genetic alteration.**(a) The scatter plot exhibited the relationship between Rnd1 expression and copy number variation in the TCGA dataset. n=396. (b) The relationship among Rnd1 expression, copy number variation, promoter methylation, somatic mutation. n=396.
