# Supplementary material for: The Rho GTPase Rnd1 inhibits epithelial–mesenchymal transition in hepatocellular carcinoma and is a favorable anti-metastasis target
Source: Cell Death Dis. 2018 Apr 30;9(5):486. doi: 10.1038/s41419-018-0517-x (PMC5924761; doi:10.1038/s41419-018-0517-x)
Supplement: Supplementary file 2 — Supplementary table 1 [file 41419_2018_517_MOESM2_ESM.docx]

**Supplementary table1. Correlations of Rnd1 expression with the clinicopathological features of HCC**

| **Clinicopathological Variables** | **n** | **Rnd1 Expression** | | ***P* value** |
| --- | --- | --- | --- | --- |
|  |  | **Low expression(17)** | **High expression(50)** |  |
| **Sex** |  |  |  |  |
| Male | 58 | 13 | 45 | 0.216 |
| Female | 9 | 4 | 5 |  |
| **Age, years** |  |  |  |  |
| ≤60 | 53 | 13 | 40 | 0.740 |
| >60 | 14 | 4 | 10 |  |
| **HBV** |  |  |  |  |
| Negative | 8 | 2 | 6 | 0.979 |
| Positive | 59 | 15 | 44 |  |
| **Liver cirrhosis** |  |  |  |  |
| Absence | 8 | 1 | 7 | 0.669 |
| Presence | 59 | 16 | 43 |  |
| **Tumor size, cm** |  |  |  |  |
| ≤5cm | 47 | 8 | 39 | **0.029** |
| ＞5cm | 20 | 9 | 11 |  |
| **AFP,** **ng/mL** |  |  |  |  |
| <20 | 27 | 8 | 19 | 0.574 |
| ≥20 | 40 | 9 | 31 |  |
| **Capsulation formation** |  |  |  |  |
| Absence | 31 | 9 | 22 | 0.581 |
| Presence | 36 | 8 | 28 |  |
| **Microvascular invasion** |  |  |  |  |
| Presence | 12 | 7 | 5 | **0.008** |
| Absence | 55 | 10 | 45 |  |
| **Edmondson-Steiner grade** |  |  |  |  |
| I & II | 43 | 7 | 36 | **0.039** |
| III & IV | 24 | 10 | 14 |  |
| **BCLC stage** |  |  |  |  |
| 0 & A | 9 | 1 | 8 | 0.430 |
| B & C | 58 | 16 | 42 |  |
| **TNM stage** |  |  |  |  |
| Early (I & II) | 59 | 16 | 43 | 0.669 |
| Late (III & IV) | 8 | 1 | 7 |  |
